# A Conserved Basal Transcription Factor Is Required for the Function of Diverse TAL Effectors in Multiple Plant Hosts

**DOI:** 10.3389/fpls.2017.01919

**Published:** 2017-11-07

**Authors:** Renyan Huang, Shugang Hui, Meng Zhang, Pei Li, Jinghua Xiao, Xianghua Li, Meng Yuan, Shiping Wang

**Affiliations:** National Key Laboratory of Crop Genetic Improvement, National Center of Plant Gene Research (Wuhan), Huazhong Agricultural University, Wuhan, China

**Keywords:** bacterial disease, basal transcription factor, transcription activator-like effector, crop, *Xanthomonas*

## Abstract

Many *Xanthomonas* bacteria use transcription activator-like effector (TALE) proteins to activate plant disease susceptibility (*S*) genes, and this activation contributes to disease. We recently reported that rice basal transcription factor IIA gamma subunit, OsTFIIAγ5, is hijacked by TALE-carrying *Xanthomonas oryzae* infecting the plants. However, whether TFIIAγs are also involved in TALE-carrying *Xanthomonas*-caused diseases in other plants is unknown. Here, molecular and genetic approaches were used to investigate the role of TFIIAγs in other plants. We found that TFIIAγs are also used by TALE-carrying *Xanthomonas* to cause disease in other plants. The TALEs of *Xanthomonas citri* pv. *citri* (*Xcc*) causing canker in citrus and *Xanthomonas campestris* pv. *vesicatoria* (*Xcv*) causing bacterial spot in pepper and tomato interacted with corresponding host TFIIAγs as in rice. Transcriptionally suppressing *TFIIAγ* led to resistance to *Xcc* in citrus and *Xcv* in pepper and tomato. The 39th residue of OsTFIIAγ5 and citrus CsTFIIAγ is vital for TALE-dependent induction of plant *S* genes. As mutated OsTFIIAγ5^V 39E^, CsTFIIAγ^V 39E^, pepper CaTFIIAγ^V 39E^, and tomato SlTFIIAγ^V 39E^ also did not interact with TALEs to prevent disease. These results suggest that TALE-carrying bacteria share a common mechanism for infecting plants. Using TFIIAγ^V 39E^-type mutation could be a general strategy for improving resistance to TALE-carrying pathogens in crops.

## Introduction

Many *Xanthomonas* bacteria use transcription activator-like effector (TALE) proteins to induce transcription of host disease susceptibility (*S*) genes, which can cause devastating diseases in plants (Zhang et al., 2015). The TALEs are the largest family of type III effectors in *Xanthomonas*, a particularly widespread phytobacterial genus consisting of almost 30 species that cause disease in more than 400 plant species, including 11 monocotyledonous and 57 dicotyledonous families (Ryan et al., 2011). Each TALE typically has a modular architecture containing an N-terminal secretion signal that guides the protein’s translocation into host cells through a type III secretion system; a central repeat domain that binds to the effector binding element (EBE) of the host gene promoter via its repeat variable di-residues (RVDs); a newly identified transcription factor binding (TFB) motif that interacts with the host basal transcription factor IIA gamma subunit (TFIIAγ); three nuclear localization signals that guide the protein’s translocation into the host nucleus; and a highly conserved C-terminal acidic activation domain that permits the TALE protein to activate transcription (Doyle et al., 2013; Zhang et al., 2015; Yuan et al., 2016).

*Xanthomonas oryzae* pv. *oryzae* (*Xoo*) and *Xanthomonas oryzae* pv. *oryzicola* (*Xoc*) cause bacterial blight and bacterial streak diseases in rice (*Oryza sativa*), respectively. A recent study revealed that the binding of *Xoo* and *Xoc* TALEs to rice EBEs via their RVDs alone cannot efficiently induce rice *S* gene expression to cause disease. The TFB motif of TALEs is vital for *Xoo* and *Xoc* to successfully invade plant cells (Yuan et al., 2016). *Xoo* and *Xoc* TALEs directly interact with rice TFIIAγ (OsTFIIAγ5) via their TFB motif to activate the transcription of rice *S* genes and enable colonization. The attenuation or absence of this interaction could eliminate or weaken the activation of rice *S* gene expression (Yuan et al., 2016). For example, rice recessive disease resistance gene *xa5*, which encodes a mutated OsTFIIAγ5 in which valine (V) is changed to glutamic acid (E) at the 39th amino acid residue (OsTFIIAγ5^V 39E^), confers broad-spectrum resistance to both *Xoo* and *Xoc* because most TALEs interact either weakly or not at all with OsTFIIAγ5^V 39E^. Partial suppression of Os*TFIIAγ5* expression also results in resistance to *Xoo* and *Xoc* (Yuan et al., 2016). Further, the rice carrying OsTFIIAγ5^V 39E^ is indistinguishable from carrying OsTFIIAγ5 in plant development and yield.

At least eight other *Xanthomonas* species [*Xanthomonas campestris* pv. *musacearum, Xanthomonas axonopodis* pv. *manihotis, Xanthomonas citri* subsp. *citri* (*Xcc*), *Xanthomonas campestris* pv. *malvacearum, Xanthomonas campestris* pv. *campestris, Xanthomonas campestris* pv. *vesicatoria* (*Xcv*), *Xanthomonas axonopodis* pv. *vasculorum*, and *Xanthomonas translucens*] also use TALEs to infect plants, including banana, cassava, citrus, cotton, brassicaceae, pepper, tomato, sugarcane, or cereal species, with severe adverse effects on crop yield and quality (Schornack et al., 2013; Boch et al., 2014). In addition to the *Xanthomonas* species, the plant bacterium *Ralstonia solanacearum*, the endosymbiont *Burkholderia rhizoxinica*, and unclassified marine microorganisms also carry TALEs that may also have a role in host-microbe interactions (de Lange et al., 2013, 2015; Juillerat et al., 2014).

TFIIA is a conserved transcription factor of eukaryotes, and it is involved in RNA polymerase II-dependent transcription. It contains two subunits, the large subunit TFIIAαβ and the small subunit TFIIAγ, encoded by separate genes (Orphanides et al., 1996). TFIIAγs from different species have high amino acid sequence similarity (Yuan et al., 2016). TFIIA interacts with other transcriptional regulatory proteins to form a transcription pre-initiation complex, which binds to the TATA-box of promoters and initiates gene expression (Høiby et al., 2007). However, it is unknown whether other TALE-carrying bacteria also hijack their corresponding host TFIIAγ to cause plant disease.

To investigate whether host TFIIAγs are required for TALE-carrying bacteria to infect other crops, we analyzed the interactions between TFIIAγs of citrus, pepper and tomato and TALEs from *Xcc*, which causes canker disease in citrus, and *Xcv*, which causes bacterial leaf spot disease in pepper and tomato. We also analyzed the importance of citrus, pepper, and tomato TFIIAγs in diseases caused by *Xcc* and *Xcv*. The results suggest that *Xcc* and *Xcv* TALEs also interact with plant TFIIAγs via their TFB motifs. The 39th amino acid residue of TFIIAγs is essential for the interaction between TFIIAγs and TALE TFB motifs. Suppressing the expression of citrus TFIIAγ gene significantly improves resistance to *Xcc*, which is associated with compromised induction of host *S* gene. Suppressing the expression of pepper and tomato TFIIAγ genes also improves resistance to *Xcv* in these plants. Therefore, our results suggest that TALE-carrying bacteria use a common mechanism to hijack host basal transcription factor for successful infection.

## Materials and Methods

### Plant and Bacterial Materials

Indica rice (*O. sativa* ssp. *indica*) IR24 and IRBB5 are near-isogenic lines. IR24 carries Os*TFIIAγ5* gene and is susceptible to *Xoo* and *Xoc*. IRBB5, with the genetic background of IR24, carries recessive disease resistance gene *xa5* encoding a mutated OsTFIIAγ5 (OsTFIIAγ5^V 39E^) and is resistant to *Xoo* and *Xoc*. Plants were grown during a normal rice-growing season under natural field conditions. The wild *Oryza* species used in this study were from our collection (Yuan et al., 2014). Sweet orange (*Citrus sinensis* L. Osbeck cv. Newhall) plants were grown in a greenhouse at 25 to 30°C. Pepper (*Capsicum annuum* L. cv. Hua 50) and tomato (*Solanum lycopersicum* L. cv. Ailsa Craig) plants were germinated and grown in a growth chamber under a photosynthetic photo flux density of approximately 120 μmol photons m^-2^ s^-1^ with a 16-h light/8-h dark photoperiod cycle at 25°C and a relative humidity of approximately 60%.

The Philippine *Xoo* strains PXO99 and PXO341 have commonly been used in studies of rice resistance to bacterial blight disease (Li et al., 2012). The *Xoc* strain RH3 is also frequently used in research (Yuan et al., 2016). *Xcc* strain X02-007 (Fu et al., 2012) and *Xcv* strain 23-1 (Sun et al., 1999) were also previously used in research. All the *Xanthomonas* strains were grown at 28°C on nutrient agar medium. When genetic manipulation of bacteria was undertaken, antibiotics were used at the following final concentrations as required: ampicillin at 100 μg ml^-1^, rifampicin at 75 μg ml^-1^, and kanamycin at 25 μg ml^-1^.

### Transformation

For stable transformation of rice, *Agrobacterium-*mediated transformation was performed using calli derived from mature embryos of indica rice line IRBB5 according to previously reported methods (Ge et al., 2006). For construction of *P_OsTFIIAγ5_:OsTFIIAγ5* derivative vectors, the 2055-bp promoter, 321-bp full-length cDNA and 1229-bp terminator of Os*TFIIAγ5* were amplified with primers listed in Supplementary Table 1 and inserted into vector pCAMBIA1301 in order. For construction of *P_Ubi_:OsTFIIAγ5* derivative vectors, 321-bp full-length cDNA of Os*TFIIAγ5* and its derivatives were amplified with primers listed in Supplementary Table 1 and inserted into vector pU1301 (Yuan et al., 2010).

For transiently suppressing the target gene in sweet orange, *Xcc*-facilitated agroinfiltration was performed. In brief, a 162-bp cDNA fragment of Cs*TFIIAγ* was amplified with primers listed in Supplementary Table 1 and inserted into vector pDS1301, and the construct was then transformed into *Agrobacterium tumefaciens* strain GV3101 (Yuan et al., 2010). Sweet orange young leaves were inoculated with *Xcc* [5 × 10^8^ colony-forming unit (cfu)/ml] by the penetration method using a needleless syringe. Eight hours later, the same inoculated leaf areas were subjected to agroinfiltration with *Agrobacterium* carrying the recombinant construct (Hu et al., 2014; Jia and Wang, 2014). The *Xcc*-facilitated agroinfiltration plants were grown in a greenhouse at 25 to 30°C with a 16-h light/8-h dark photoperiod cycle.

For transiently suppressing target gene in pepper and tomato, virus-induced gene silencing (VIGS) was performed. In brief, the DNA segment of the target gene or green fluorescence protein (GFP) gene was inserted into the tobacco rattle virus (TRV) vector pTRV2 as described previously (Liu et al., 2002a). The recombinant vectors were introduced into GV3101. *Agrobacterium* culture was infiltrated into the cotyledons of germinating pepper plants or the first two true leaves of the four-leaf stage tomato plants using a 1-ml needleless syringe (Liu et al., 2002b; Chung et al., 2004). The *Agrobacterium-*infiltrated plants were first kept in a growth chamber at 16°C for 1 day and then grown in a chamber at 25°C with a 16-h light/8-h dark photoperiod cycle.

### Protein–Protein Interaction

To study the interaction between TALE and host proteins in yeast two-hybrid assays (Yuan et al., 2010), the TFB motif of TALE genes and plant *TFIIAγ* genes were amplified using PCR primers listed in Supplementary Table 1. The amplified bacterial DNA segments were ligated into pGBKT7 vector, and the amplified plant DNA segments were ligated into pGADT7 Rec vector. The pGBKT7 and pGADT7 plasmids were then transformed into yeast strain AH109 (Yuan et al., 2010). The yeast clones were scribed on the synthetic defined premixes (SD) medium lacking leucine (L) and tryptophan (W) (-LW) and selective SD medium lacking L, W, histidine (H), and adenine (A) (-LWHA). The interactions of examined proteins were assessed by growth of yeast cells on selective medium and by examination of β-D-galactopyranoside (X-α-gal) activity and β-galactosidase (LacZ) activity as described previously (Yuan et al., 2010).

To study the interaction between TALE and plant proteins in planta, co-immunoprecipitation (CoIP) assays were performed (Yuan et al., 2016). The DNA segments of TFB motifs of TALEs were ligated into the pU1031-9myc vector, and the DNA segments of plant genes were ligated into the pU1301-3FLAG vector (Yuan et al., 2010). The recombinant vectors were introduced into GV3101. *Agrobacterium*-mediated transformation was performed by infiltrating into *Nicotiana benthamiana* leaves using a needleless syringe. CoIP assays were conducted using anti-FLAG antibody (Sigma-Aldrich, St. Louis, MO, United States) and anti-myc antibody (Tiangen, Beijing, China) as described previously (Yuan et al., 2016). Each CoIP assay was repeated at least twice. The original western blotting images are provided in Supplementary Figure 13.

### Site-Directed Mutation

Mutation of plant genes was performed using the GeneTailor Site-Directed Mutagenesis System (Invitrogen Life Technologies, Carlsbad, CA, United States) as described previously (Yuan et al., 2011). The mutagenic primers are listed in Supplementary Table 1.

### Pathogen Inoculation

To evaluate rice bacterial blight disease, rice plants were inoculated with *Xoo* by the leaf-clipping method at the booting (panicle development) stage (Chen et al., 2002). Disease was scored by measuring the lesion length at 14 days after inoculation.

To evaluate rice bacterial streak disease, rice plants were inoculated with *Xoc* strains by the needle stab method at the tillering stage (Yuan et al., 2016). The disease was scored by measuring the lesion length at 14 days after inoculation.

To evaluate citrus bacterial canker disease, sweet orange leaves were inoculated with *Xcc* at a concentration of 5 × 10^8^ cfu/ml in 10 mM MgCl_2_ using a needleless syringe. The bacterial growth rate in sweet orange leaves was measured by counting the cfu as described previously (Hu et al., 2014).

To evaluate bacterial spot disease of pepper and tomato, 4-week-old VIGS-treated plants were sprayed with *Xcv* at a concentration of 5 × 10^5^ cfu/ml in 10 mM MgCl_2_. The bacterial growth rate in pepper or tomato leaves was measured by counting the cfu as described previously (Bonas et al., 1991).

### Gene Expression Analysis

For gene expression analysis, 2-cm rice leaf fragments near the bacterial infection sites or citrus, pepper and tomato leaf tissues next to the infiltration sites were collected for RNA isolation. Quantitative reverse transcription-PCR (qRT-PCR) was conducted using gene-specific primers (Supplementary Table 2) as described previously (Yuan et al., 2010). The expression level of rice, pepper or tomato *actin* gene or citrus *EF1a* gene was used to standardize the RNA sample of rice, pepper, tomato or citrus, respectively. The expression level relative to that of controls was assessed. Each qRT-PCR assay was repeated at least twice with similar result, with each repetition having three replicates.

### Statistical Analysis

Differences between samples were analyzed for statistical significance by using the SPSS software and the Student’s *t*-test (two tailed).

## Results

### *Xcc* Uses Host TFIIAγ to Cause Disease in Sweet Orange

**FIGURE 1:**
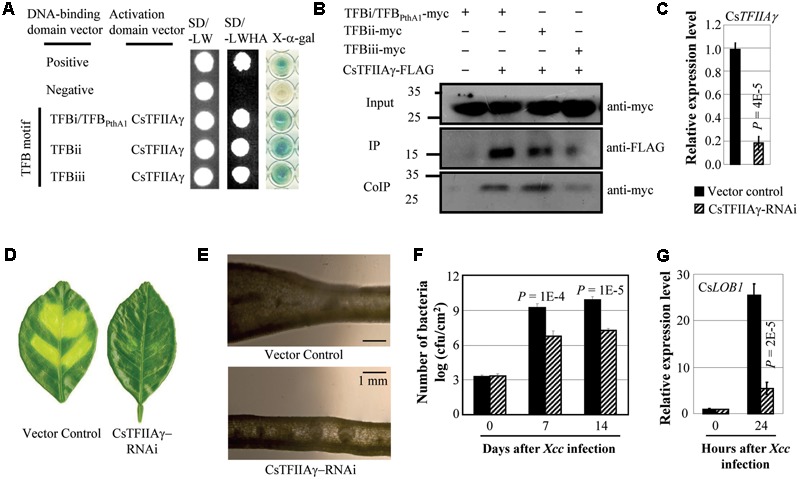
Effect of CsTFIIAγ in TALE-carrying bacterium *Xcc* caused infection of sweet orange. **(A)** The TFB motifs of all three TALEs from *Xcc* strain X02-007 interacted with CsTFIIAγ analyzed using yeast two-hybrid assay. Among the three TFB motifs, the sequence of TFBi is the same as the TFB motif of TALE PthA1 from sequenced *Xcc* strain 306. The interactions were assessed by growth of yeast cells on synthetic defined premixes (SD) medium lacking leucine (L), tryptophan (W), histidine (H), and adenine (A). **(B)** The myc-labeled TFBs from *Xcc* interacted with FLAG-labeled CsTFIIAγ in *Nicotiana benthamiana* leaf cells analyzed by co-immunoprecipitation (CoIP) assay. Proteins before (input) and after immunoprecipitation (IP) were detected with anti-myc and anti-FLAG antibodies. **(C)** Expression of Cs*TFIIA*γ in transgenic and control plants. **(D)** The Cs*TFIIA*γ-RNAi plants showed resistance to *Xcc* 14 days after bacteria inoculation. **(E)** Thin cross-section images of leaves at 14 days after inoculation with *Xcc*. **(F)** Cs*TFIIAγ*-RNAi plants had less *Xcc* growth. Bar represents mean (three replicates) ± standard deviation. cfu, colony-forming unit. **(G)** Expression of disease susceptibility gene *CsLOB1* after infection of *Xcc*. The corresponding *P*-values were determined using Student’s *t*-test (two tailed) comparing data from the control and suppressing plants.

*Xcc* causes citrus canker. *Xcc* strain X02-007 carries TALE genes, which contained TFB motifs of TALEs that showed high sequence similarity with the motifs of *Xoo* and *Xoc* TALEs (Supplementary Figure 1; Yuan et al., 2016). The TFB motifs of *Xcc* TALEs interacted with sweet orange TFIIAγ (CsTFIIAγ) both in yeast and *in planta* (**Figures 1A,B**). To investigate whether CsTFIIAγ is required for TALE-dependent induction of the host *S* gene during *Xcc* invasion, we suppressed Cs*TFIIAγ* gene expression in the leaves of sweet orange by RNA interference (RNAi). The plants with suppressed expression of Cs*TFIIAγ* appeared markedly less diseased, with less cell division and proliferation or hyperplasia after *Xcc* inoculation (**Figures 1C–E**) and significantly lower (*P* < 0.01) *Xcc* growth rate compared to control plants (**Figure 1F**). Citrus Cs*LOB1* is a *S* gene to *Xcc* (Hu et al., 2014; Li et al., 2014). Consistent with reduced disease symptoms, the CsTFIIAγ-RNAi leaves showed significantly suppressed *Xcc*-induced expression of Cs*LOB1* compared with control (**Figure 1G**).

Further, different citrus species, such as hongkong kumquat, grapefruit and trifoliate orange, have a TFIIAγ protein sequence identical to CsTFIIAγ in sweet orange, although the gene sequences differ for a few nucleotides (Supplementary Figure 2A). All the citrus species were similarly susceptible to *Xcc* (Supplementary Figure 2B). These results suggest that, like OsTFIIAγ5, CsTFIIAγ is also hijacked by TALE-carrying bacteria, with the host becoming infected via TALE-induced expression of the host *S* gene.

### The 39th Amino Acid Residue of Plant TFIIAγs Is Essential for the Virulence of TALE-Carrying Bacteria

Rice plants carrying recessive *xa5*, which encodes the mutated OsTFIIAγ5^V 39E^, have broad-spectrum resistance to TALE-carrying bacteria and no effect on rice yield and development (Yuan et al., 2016). To analyze whether OsTFIIAγ5^V 39E^ is the only type of mutation that confers disease resistance in rice and whether mutations similar to OsTFIIAγ5^V 39E^ can confer disease resistance in other plant species, we first examined the sequences of OsTFIIAγ5 coding region in different *Oryza* species.

The *Oryza* genus consists of two cultivated rice species, Asian cultivated rice (*O. sativa*, AA genome) and African cultivated rice (*O. glaberrima*, AA genome), and wild (undomesticated) rice species (AA, BB, CC, BBCC, CCDD, EE, FF, GG, HHJJ, KKLL genomes) (Ge et al., 1999; Ammiraju et al., 2010). We sequenced the Os*TFIIAγ5* locus in 15 different *Oryza* genomes, including nine accessions of AA genome (*O. sativa indica, O. sativa japonica, O. rufipogon, O. nivara, O. glaberrima, O. longistaminata, O. meridionalis, O. gluaepatula*, and *O. barthii*), one accession of BB genome (*O. punctata*), two accessions of CC genome (*O. officinalis* and *O. rhizomatis*), one accession of EE genome (*O. australiensis*), one accession of FF genome (*O. brachyantha*), and one accession of GG genome (*O. meyeriana*). Sequence alignment analysis showed that different *Oryza* species contained the same sequences of the Os*TFIIAγ5* coding region and encoded the identical OsTFIIAγ5 protein, which is consistent with its molecular function as a basal transcription factor.

We searched the putative OsTFIIAγ5 variant from database OryzaGenome^1^, which contains genotype information of 463 accessions of wild rice *O. rufipogon*, which is evolutionally closely related to cultivated rice (Ohyanagi et al., 2016). Thirty-four single nucleotide polymorphisms (SNPs) were distributed in the Os*TFIIAγ5* gene region, all within the introns and none in the exons. Thus the 463 *O. rufipogon* accessions encode the identical OsTFIIAγ5 protein. Moreover, we also searched the putative OsTFIIAγ5 variants from Rice SNP-Seek Database^2^, which contain genotype information of 3024 *O. sativa* varieties from the International Rice GenBank Collection Information System (IRGCIS^3^). There were 93 SNPs distributed in the 6260-bp region of Os*TFIIAγ5* gene, and 105 rice varieties contained SNPs in the coding region causing amino acid mutation. Of these 105 rice varieties, 104 have a mutated Os*TFIIAγ5* gene encoding OsTFIIAγ5^V 39E^, identical to the protein encoded by recessive disease resistance gene *xa5*. One variety has a different type of mutated Os*TFIIAγ5* gene, which encodes a protein with valine changed to aspartic acid (D) at the 39th residue (OsTFIIAγ5^V 39D^) (Supplementary Table 3). Eighty-five of the 104 rice varieties carrying the mutated Os*TFIIAγ5^V 39E^* gene and one carrying the mutated Os*TFIIAγ5^V 39D^* gene belong to the Aus group, which is mainly from South Asia, and the other 19 carrying the mutated Os*TFIIAγ5^V 39E^* gene belong to Indica II, Indica IB, Indx and Admix groups, mainly from Southeast Asia (Huang et al., 2011). The distribution of Os*TFIIAγ5^V 39E^* resistance allele in these groups likely reflects the high disease pressure in these regions, where *Xoo* is a devastating disease. The results suggest that the Os*TFIIAγ5* gene mutation probably appeared in cultivated rice.

Amino acid sequence alignment of TFIIAγs from different species showed that they share a high sequence similarity, with the 39th amino acid residue having three types, V, leucine (L), and E (Yuan et al., 2016). The V type occurs in plants (*O. sativa, Arabidopsis thaliana, Citrus sinensis, Capsicum annuum*, and *Solanum lycopersicum*). The L type occurs in animals (*Rattus norvegicus, Homo sapiens, Danio rerio*, and *Drosophila melanogaster*). The E type was only detected in rice Os*TFIIAγ5* locus (OsTFIIAγ5^V 39E^), which is recessively inherited. In addition, we identified a D type in Os*TFIIAγ5* locus (OsTFIIAγ5^V 39D^) from rice germplasm (Supplementary Table 3). To determine whether the 39th amino acid of OsTFIIAγ5 is vital for TALE-dependent induction of rice genes, we first produced four OsTFIIAγ5 derivatives at the 39th residue, substituting V with alanine (A), D, L or glutamine (Q); V, A and L are hydrophobic amino acids, and D and Q are acidic hydrophilic amino acids. The TFB motifs of TALE PthXo1 from *Xoo* and TALE Tal3c from *Xoc* interacted as strongly with mutants OsTFIIAγ5^V 39A^, OsTFIIAγ5^V 39D^, OsTFIIAγ5^V 39L^, and OsTFIIAγ5^V 39Q^ as with wild-type OsTFIIAγ5 in yeast cells (Supplementary Figure 3). The PthXo1 TFB interacted weakly and Tal3c TFB did not interact with OsTFIIAγ5^V 39E^ (E being an acidic hydrophilic amino acid) as reported previously (Yuan et al., 2016).

**FIGURE 2:**
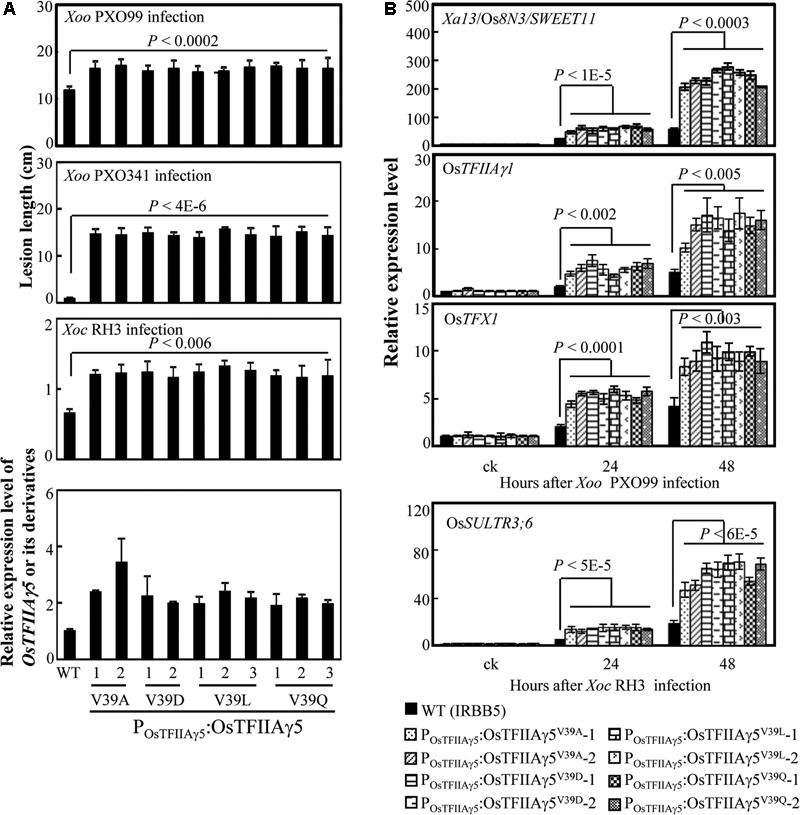
Effects of Os*TFIIAγ5* mutants in rice responses to *Xoo* and *Xoc.* **(A)** Transgenic rice plants carrying Os*TFIIAγ5* mutant were susceptible to *Xoo* stains PXO99 and PXO341 and *Xoc* strain RH3 compared to wild-type (WT) IRBB5, which carries the mutated Os*TFIIA*γ5^V 39E^. T1 plants were inoculated with *Xoo* at the booting stage and *Xoc* at the tillering stage. Data represent mean (total 20 to 25 leaves from four plants) ± standard deviation. **(B)** Expression of disease susceptibility genes *Xa13/Os8N3/SWEET11*, Os*TFIIAγ1*, and Os*TFX1* in rice after infection of PXO99 and *OsSULTR3;6* after infection of RH3. Bar represents mean (three replicates) ± standard deviation. The corresponding *P*-values were determined using Student’s *t*-test (two tailed) comparing data from the WT and transgenic plants.

We then generated Os*TFIIAγ5* transgenic plants with the four different amino acid substitutions (A, D, L, and Q) at the 39th residue driven by the native promoter of Os*TFIIAγ5* (*P_OsTFIIAγ5_*) in the *xa5* (Os*TFIIAγ5^V 39E^*) resistance allele background (IRBB5). The P_OsTFIIAγ5_:OsTFIIAγ5^V 39A^, P_OsTFIIAγ5_:OsTFIIAγ5^V 39D^, P_OsTFIIAγ5_:OsTFIIAγ5^V 39L^, and P_OsTFIIAγ5_:OsTFIIAγ5^V 39Q^ plants showed susceptibility to *Xoo* strains PXO99 and PXO341, also susceptibility to *Xoc* strain RH3 compared to the wild-type plant (**Figure 2A**). The susceptibility of these transgenic plants to *Xoo* was associated with more efficiently induced expression of known rice *S* genes (*Xa13*/Os*8N3*/*SWEET11*, Os*TFIIAγ1*, and Os*TFX1*) to *Xoo* (**Figure 2B**; Chu et al., 2006; Sugio et al., 2007). The susceptibility of these transgenic plants to *Xoc* was associated with more efficiently induced expression of the known rice *S* gene (*OsSULTR3;6*) to *Xoc* (**Figure 2B**; Cernadas et al., 2014). We also generated Os*TFIIAγ5* overexpressing plants with the same mutations listed above driven by maize ubiquitin promoter (*P_Ubi_*). These transgenic plants also showed increased susceptibility to *Xoo* and *Xoc*, which was associated with increased induced expression of rice susceptibility genes, compared to wild-type plants (Supplementary Figures 4, 5). These results suggest that the 39th amino acid residue of OsTFIIAγ5 is essential for TALE-dependent induction of rice *S* genes, and it seems that only the V to E mutation at this residue (OsTFIIAγ5^V 39E^) affects the interaction between OsTFIIAγ5 and TFB of TALEs.

**FIGURE 3:**
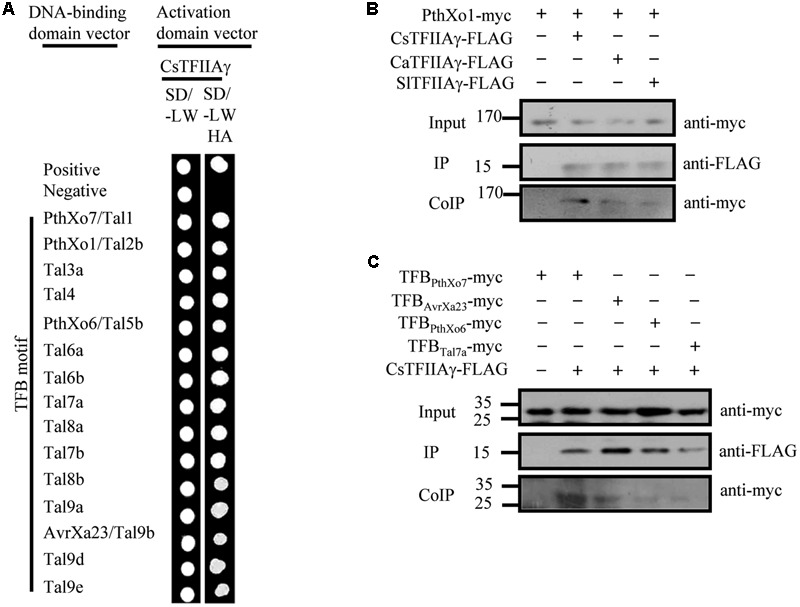
Analysis of the interactions between *Xoo* TALEs and plant TFIIAγs (citrus CsTFIIAγ, pepper CaTFIIAγ, and tomato SlTFIIAγ). **(A)** Interactions between TFB motif of *Xoo* TALEs and CsTFIIAγ as determined by yeast two-hybrid. **(B)** Interactions of the myc-labeled full-length TALE PthXo1 with FLAG-labeled plant TFIIAγs in *N. benthamiana* leaf cells by CoIP. **(C)** Interaction of the myc-labeled TFB motif of four TALEs with FLAG-labeled plant TFIIAγs in *N. benthamiana* leaf cells by CoIP.

The 39th amino acid residue of citrus CsTFIIAγ is also V, and CsTFIIAγ and OsTFIIAγ5 share 93/89% sequence similarity/identity (Supplementary Figure 6; Yuan et al., 2016). Furthermore, the TFB motifs of *Xcc* TALEs have high sequence homology with TFBs of *Xoo* and *Xoc* TALEs (Supplementary Figure 1). To investigate whether the OsTFIIAγ5^V 39E^ type mutation in CsTFIIAγ (CsTFIIAγ^V 39E^) could prevent TALE-carrying bacteria from infecting plants, we first examined the interactions between the TALE TFB motifs of different *Xanthomonas* bacteria and CsTFIIAγ^V 39E^. The TFB motifs of *Xoo, Xoc*, and *Xcc* TALEs could not interact with CsTFIIAγ^V 39E^ (Supplementary Figures 7A, 8A), although these TFBs interacted strongly with both OsTFIIAγ5 and CsTFIIAγ in yeast cells and *in planta* (**Figures 1, 3** and Supplementary Figure 7B). The only exception was the TFB motif of *Xoo* Tal7a. It interacted with CsTFIIAγ^V 39E^ (Supplementary Figure 7A), which is consistent with this TFB also interacting with OsTFIIAγ5^V 39E^ (Yuan et al., 2016).

**FIGURE 4:**
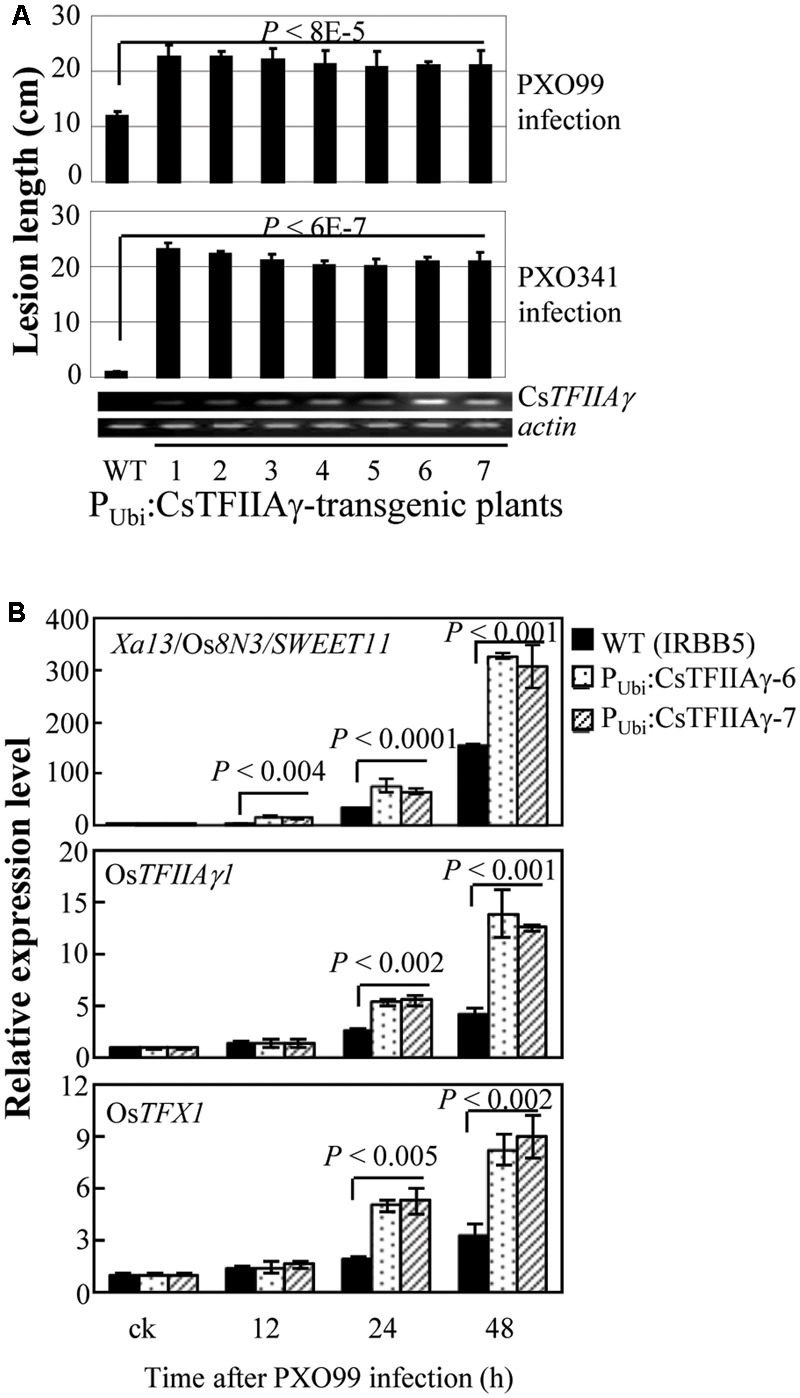
Effect of overexpressing *CsTFIIAγ* (P_Ubi_:CsTFIIAγ) in rice response to *Xoo.* Plants were inoculated with *Xoo* strains PXO99 and PXO341 at the booting (panicle development) stage. **(A)** Transgenic rice plants (T1 generation) were susceptible to *Xoo* compared to WT. Data represent mean (5 to 10 replicates from one plant) ± standard deviation. RT-PCR analysis of *CsTFIIAγ* transcripts in transgenic plants. *Actin* transcripts were detected as control. **(B)** Expression of disease susceptibility genes *Xa13/Os8N3/SWEET11*, Os*TFIIAγ1*, and Os*TFX1* in rice after infection of *Xoo* strain PXO99. Bar represents mean (three replicates) ± standard deviation. The corresponding *P*-values were determined using Student’s *t*-test (two tailed) comparing data from the WT and transgenic plants.

Furthermore, we overexpressed Cs*TFIIAγ* in rice IRBB5 carrying the recessive *xa5* gene. The transgenic plants displayed susceptibility to *Xoo* strains PXO99 and PXO341 (**Figure 4A**). The susceptibility was associated with overexpression of Cs*TFIIAγ*, which was further confirmed in two independent T1 families (Supplementary Figure 9). The induced expression of known *S* genes *Xa13/Os8N3/SWEET11, OsTFIIAγ1*, and *OsTFX1*, each of which is targeted by a different TALE, was significantly higher (*P* < 0.01) in transgenic plants than in wild-type plants (**Figure 4B**). Similarly, overexpressing Cs*TFIIAγ* in rice increased susceptibility to *Xoc*, which was associated with significantly promoted *Xoc*-induced expression of rice *S* gene *OsSULTR3;6* compared to wild-type plants (Supplementary Figure 10). However, the transgenic plants with overexpression of Cs*TFIIAγ^V 39E^* showed similar lesion length compared to wild-type plants upon inoculation with *Xoo* strain PXO99 (Supplementary Figure 11). These results suggest that CsTFIIAγ can also facilitate the infection of *Xoo* and *Xoc* in rice by helping TALE-induced activation of rice *S* genes, and the 39th amino acid residue of CsTFIIAγ is also essential for TALE-dependent induction of host *S* genes.

### *Xcv* Also Uses Host TFIIAγ to Cause Disease in Pepper and Tomato

**FIGURE 5:**
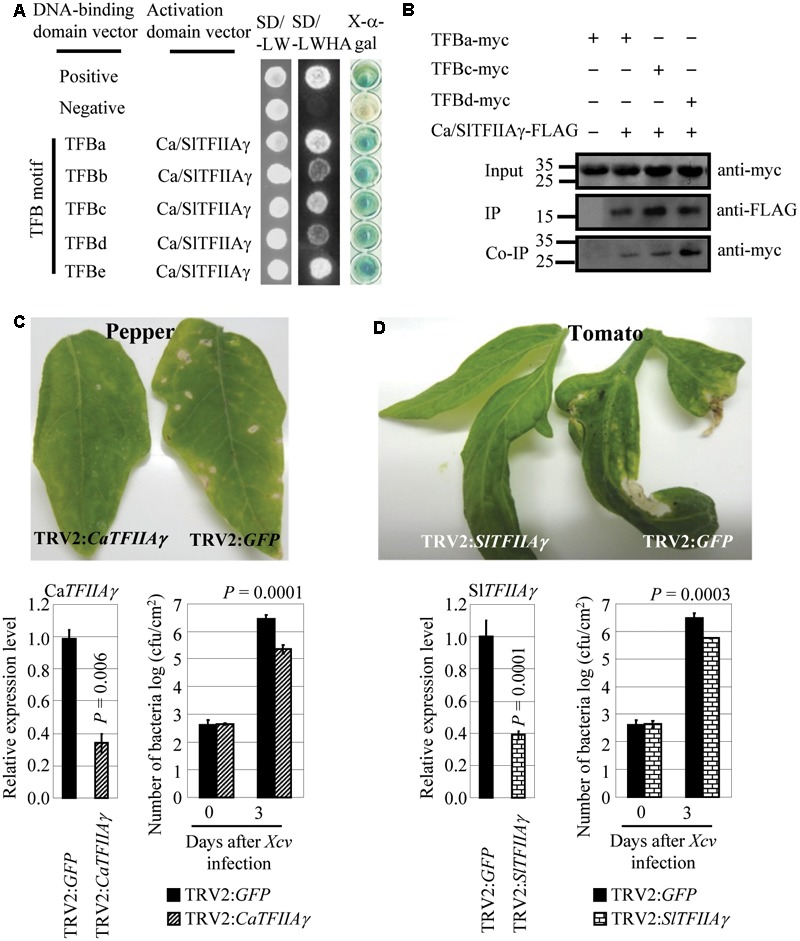
Effects of CaTFIIAγ and SlTFIIAγ in TALE-carrying bacterium *Xcv*-caused infection of pepper and tomato. **(A)** The TFB motifs of all the five TALEs from *Xcv* strain 23-1 interacted with CaTFIIAγ and SlTFIIAγ analyzed using yeast two-hybrid assay. **(B)** The myc-labeled TFB motifs of *Xcv* TALEs interacted with FLAG-labeled CaTFIIAγ and SlTFIIAγ in *N. benthamiana* leaf cells analyzed by CoIP. Proteins before (input) and after immunoprecipitation (IP) were detected with anti-myc and anti-FLAG antibodies. **(C)** Suppressing Ca*TFIIAγ* enhanced pepper resistance to *Xcv*. Bar represents mean (three replicates) ± standard deviation. cfu, colony-forming unit. **(D)** Suppressing Sl*TFIIAγ* enhanced tomato resistance to *Xcv*. Bar represents mean (three replicates) ± standard deviation. The corresponding *P*-values were determined using Student’s *t*-test (two tailed) comparing data from the control and suppressing plants.

We also examined the relationship between pepper (*Capsicum annuum* L. cv. Hua 50) CaTFIIAγ or tomato (*Solanum lycopersicum* L. cv. Ailsa Craig) SlTFIIAγ and *Xcv*, which causes bacterial spot disease in the two crops. CaTFIIAγ and SlTFIIAγ have identical sequences, and *Xcv* strain 23-1 carries TALE genes (Yuan et al., 2016). The TFB motifs of *Xcv* 23-1 TALEs, which featured high sequence similarity with the TFB motifs of *Xoo, Xoc*, and *Xcc* TALEs (Supplementary Figure 1), interacted with CaTFIIAγ/SlTFIIAγ both in yeast and *in planta* (**Figures 5A,B**), but they did not interact with Ca/SlTFIIAγ^V 39E^ (Supplementary Figures 8B, 12). Transiently suppressing Ca*TFIIAγ* or Sl*TFIIAγ* by VIGS enhanced the resistance of pepper and tomato to *Xcv*. The pepper plants with suppressed expression of Ca*TFIIAγ* (TRV2:Ca*TFIIAγ*) and tomato plants with suppressed expression of Sl*TFIIAγ* (TRV2:Sl*TFIIAγ*) showed markedly reduced disease symptoms and significantly lower (*P* < 0.01) *Xcv* growth rates compared to corresponding control plants expressing TRV2:*GFP* (**Figures 5C,D**). These results suggest that TALE-containing *Xcv* causes diseases in pepper and tomato and may also need the help of host TFIIAγs.

## Discussion

In addition to TALE-carrying *Xoo* and *Xoc* needing the help of host TFIIAγ (OsTFIIAγ5) to cause disease in rice (Yuan et al., 2016), the present results show that TALE-carrying *Xcc* also requires host TFIIAγ (Cs*TFIIAγ*) for successful colonization in sweet orange. Transcriptional suppression of Cs*TFIIAγ* can reduce disease symptoms. *Xoo* and *Xoc* employ OsTFIIAγ5 to facilitate TALE-induced expression of host *S* genes for infection (Yuan et al., 2016). Efficient binding of the TFB motifs of *Xoo* and *Xoc* TALEs to OsTFIIAγ5 is important for the induction of *S* genes. The present results suggest that *Xcc* likely uses the same mechanism as *Xoo* and *Xoc* to infect its host. This inference is supported by the following evidence. First, the TFBs of *Xcc* TALEs also interacted with CsTFIIAγ. Second, transcriptional suppression of Cs*TFIIAγ* resulted in reduced disease of sweet orange and was associated with compromised induction of *S* gene Cs*LOB1*. Last, because of the high amino acid homology among TALE TFBs of different *Xanthomonas* species, such as *Xoo, Xoc, Xcc*, and *Xcv* (Supplementary Figure 1), and among different TFIIAγs of different eukaryote species, such as OsTFIIAγ5, CsTFIIAγ, CaTFIIAγ, and SlTFIIAγ (Yuan et al., 2016), CsTFIIAγ could be used by *Xoo* and *Xoc* to cause disease in rice, which was associated with induced expression of rice *S* genes. Although high amino acid sequence similarity was present among TALE TFBs of different *Xanthomonas* species, there was still some dissimilarity in the motif (Supplementary Figure 1; Yuan et al., 2016). Protein residues diversity may influence the interaction strength with plant TFIIAγ, which might be supported by the relatively weak band in some of the CoIP assays.

The present results also show that *Xcv* TALEs can bind to pepper CaTFIIAγ and tomato SlTFIIAγ through their TFBs. Suppression of CaTFIIAγ and SlTFIIAγ reduces *Xcv*-caused disease symptoms. Although the present study does not identify which host gene is targeted by the TALEs of *Xcv* strain 23-1, the results suggest that CaTFIIAγ and SlTFIIAγ may also be hijacked by *Xcv* for infection. This inference is also supported by the localization characteristic of the TALE binding sites (EBEs) of host gene promoters in different plants. We analyzed 45 EBEs of known *S* genes and executor resistance genes that can be directly targeted by *Xoo, Xoc, Xcv, Xcc, Xam*, or *Xg* TALEs in rice, pepper, citrus, cassava, or tomato (Supplementary Table 4). These EBEs either overlapped their corresponding TATA-box or were located close to it, and some TATA-boxes are the core part of EBEs, which is consistent with computational model-based predictions that target sites of TALEs are preferentially located at most 300 bp upstream and 200 bp downstream of the transcription start site of target genes (Grau et al., 2013). TFIIAγ is a component of a transcription pre-initiation complex that binds to the TATA-box of promoters to initiate gene expression in eukaryotes (Høiby et al., 2007). The overlap or adjacent physical position of TALE-binding sites in relation to the positions of TATA-box supports the possibility that *Xcv* TALEs also use CaTFIIAγ and SlTFIIAγ for infection in pepper and tomato. Furthermore, the localization relationship between EBE and TATA-box also suggests that other TALE-carrying bacteria, in addition to *Xoo, Xoc, Xcc*, and *Xcv*, may also require the help of host TFIIAγ for infection.

Our previous and present results indicate that the difference in interaction strength between different TFB motifs from diverse *Xanthomonas* TALEs and corresponding host plant TFIIAγ with V39E mutation could be correlated with specific differences in the amino acids of the TFB motifs (Supplementary Figures 1, 3, 7, 12). The present results undoubtedly suggest that the 39th amino acid residue of OsTFIIAγ5 is important for TALE-dependent induction of host genes. The OsTFIIAγ5^V 39E^ encoded by recessive disease resistance gene *xa5* is the only type of mutation identified so far that can prevent or attenuate the infection of *Xoo* and *Xoc* in rice (Supplementary Table 3; Yuan et al., 2016). The V39E substitution of CsTFIIAγ (CsTFIIAγ^V 39E^) also prevented its interaction with TFBs of *Xcc* TALEs and abolished or compromised CsTFIIAγ-facilitated infection of *Xoo* and *Xoc* in rice, suggesting that the CsTFIIAγ^V 39E^ mutation may make citrus resistance to *Xcc*. In addition, the TFBs of *Xcv* TALEs cannot interact with CaTFIIAγ^V 39E^ and SlTFIIAγ^V 39E^, suggesting that the TFIIAγ^V 39E^-type mutation may also be used to prevent *Xcv*-caused diseases in pepper and tomato. Moreover, TFBs with mutation in the 134 amino acids motif might be found with more *Xcc* or *Xcv* strains been sequenced, which can break CsTFIIAγ^V 39E^, CaTFIIAγ^V 39E^, and SlTFIIAγ^V 39E^ mediated resistance to *Xcc* or *Xcv*. An example is that the TFBs of pthXo1, tal7a, and tal8a of *Xoo* pv. PXO99 differed by 1 to 20 residues from the other 12 TFBs could interact with OsTFIIAγ^V 39E^, and the rice variety IRBB5 carrying OsTFIIAγ^V 39E^ is susceptible to PXO99 (Yuan et al., 2016).

In summary, the previous report (Yuan et al., 2016) and the present results suggest that TALE-carrying bacteria probably use a common mechanism that is employing host TFIIAγs to cause disease in rice, citrus, pepper, and tomato. The genus *Xanthomonas* infects a wide variety of hosts. Because of the high amino acid sequence homology among TFIIAγs from different plant species and among the TFB motifs of TALEs from different *Xanthomonas* species, other TALE-carrying *Xanthomonas* species, which have not been examined, likely also hijack host TFIIAγs for successful infection. Since rice plant OsTFIIAγ5^V 39E^ confers broad-spectrum resistance to *Xoo* and *Xoc* without affecting rice development and yield (Yuan et al., 2016) and the present results suggest that CsTFIIAγ^V 39E^ can also compromise bacterial infection, use of TFIIAγ^V 39E^-type of mutation—either by exploring germplasm for natural mutants or using gene-editing technologies—may provide a general strategy for improving resistance to *Xcv* and other TALE-carrying pathogens in crops in addition to rice and citrus.

## Additional Information

Gene sequences of *CsTFIIAγ* from different *Citrus* species have been deposited in GenBank with the following accession codes KU377725 (in *Citrus sinensis* Hongkong Kumquat), KU377726 (in *Citrus sinensis* Sweet orange), KU377727 (in *Citrus sinensis* Grapefruit), and KU377728 (in *Citrus sinensis* Trifoliate orange).

## Author Contributions

MY, RH, and SH performed most of the experiments and analyzed the data; MZ and PL helped to generate transgenic rice plants, analyze protein–protein interactions, and amplify TALE; JX and XL provided biochemical and molecular analysis support and management; MY and SW designed the research, supervised the project, interpreted data, and drafted and revised the manuscript.

## Conflict of Interest Statement

The authors declare that the research was conducted in the absence of any commercial or financial relationships that could be construed as a potential conflict of interest.

## Footnotes

1 http://viewer.shigen.info/oryzagenome/mapview/Top.do

2 http://snp-seek.irri.org/

3 http://irgcis.irri.org:81/grc/irgcishome.html
