# Supplementary material for: A Conserved Basal Transcription Factor Is Required for the Function of Diverse TAL Effectors in Multiple Plant Hosts
Source: Front Plant Sci. 2017 Nov 7;8:1919. doi: 10.3389/fpls.2017.01919 (PMC5681966; doi:10.3389/fpls.2017.01919)
Supplement: Supplementary file 1 [file Table_1.DOC]

**Supplementary Table 1.** PCR primers used for construction of vectors for transformation and protein–protein interactions, and detection of positive transgenic plants

| Gene (accession number) | Forward primer (5’-3’) | Reverse primer (5’-3’) | Product size (nt) | Use |
| --- | --- | --- | --- | --- |
| *OsTFIIAγ5/Xa5* (AK065182) | CGGGGTACC^a^ATGGCCACCTTCGAGCTCTACCGGAGG | CGCGGATCC^b^TTGGCTGAGTAGTTTGGAATCACAGG | 318 | Amplifying cDNA fragment for constructing expressing construct |
|  | CGGGGTACC^a^AAAAGATAACCCGTAGCAGTAACAAG | ACGCGTCGAC^c^GGGCGATGCGTGCGCCTAAACTTTTTGG | 2055 | Amplifying promoter for constructing expressing construct |
|  | CCCAAGCTT^d^ATTGATAACTGCGAGGTCAGGGTTTGCC | CCCAAGCTT^d^GACCTTTCGACTGGAGCTTAACTTGCAC | 1229 | Amplifying terminator for constructing expressing construct |
| *AtTFIIAγ* (NM_179103) | CGGGGTACC^a^ATGGCGACGTTTGAGCTGTACAGGAG | CGCGGATCC^b^CTGTGTGAGCAGCTTGGAATCACATG | 318 | Amplifying cDNA fragment for constructing expressing construct |
|  | CCGGAATTC^g^ATGGCGACGTTTGAGCTGTACAGGAG | CGCGGATCC^b^TTACTGTGTGAGCAGCTTGGAATCACATG | 321 | Amplifying cDNA fragment for constructing yeast two hybrid construct |
| *CsTFIIAγ* (KU377726) | CGGGGTACC^a^ATGGCGACGTTTGAGCTGTATCGC | CGCGGATCC^b^TTGTGATAGCAGCTTTGAGTCAC | 318 | Amplifying cDNA fragment for constructing expressing construct |
|  | CCGGAATTC^g^ATGGCGACGTTTGAGCTGTATCGC | CGCGGATCC^b^TTATTGTGATAGCAGCTTTGAGTCAC | 321 | Amplifying cDNA fragment for constructing yeast two hybrid construct |
|  | AGACTAGT^e^GGTACC^a^ATGGCGACGTTTGAGC | AAGAGCTC^f^GGATCC^b^CTTCACCTGAGTTTC | 162 | Amplifying cDNA fragment for constructing RNAi construct |
| *CaTFIIAγ* (KU163013) | CCGGAATTC^g^TTTGACAAGTCAATGACTG | CGCGGATCC^b^TCACTGTGTGAGCAGCTTTG | 201 | Amplifying cDNA fragment for constructing VIGS construct |
|  | CCGGAATTC^g^ATGGCGACTTTTGAGC | CGCGGATCC^b^TCACTGTGTGAGCAGCTTTG | 321 | Amplifying cDNA fragment for constructing yeast two hybrid construct |
| TFB | CCGGAATTC^g^AGCATTGTTGCCCAGTTATCTCG | CGCGGATCC^b^CATCCCTGATGCCTGGAGGATAC | 402 | Amplifying DNA fragment from different TALEs for constructing yeast two hybrid constructs |

^a^The underlined nucleotides are the digestion site of *Kpn*I.

^b^The underlined nucleotides are the digestion site of *Bam*HI.

^c^The underlined nucleotides are the digestion site of *Sal*I.

^d^The underlined nucleotides are the digestion site of *Hin*dIII.

^e^The underlined nucleotides are the digestion site of *Spe*I.

^f^The underlined nucleotides are the digestion site of *Sac*I.

^g^The underlined nucleotides are the digestion site of *Eco*RI.

**Supplementary Table 2. PCR primers used for quantitative RT-PCR assays**

| Gene (GenBank accession no.) | Forward primer (5’-3’) | Reverse primer (5’-3’) | Product size (nt) |
| --- | --- | --- | --- |
| *Xa13/*Os*8N3*  (DQ421395) | TGGTTCTGCTACGGCCTCTT | GGTACCAGAAGTAGAGCCCCATCT | 103 |
| *OsTFIIAγ5* (AK065182) | TGGCCACCTTCGAGCTCTA | CTCGTCGAGCGTCTCAGTGA | 101 |
| *OsTFIIAγ1* (CB097192) | CGAGCTCGCCATCCAAGT | TGTGCAGATGGCCCTTGAC | 101 |
| *OsTFX1* (AK108319) | CCCACTACCACAGCAACATGA | CACAGGTAGCTGCTGGGAAGT | 101 |
| *OsSULTR3;6* (AK121195) | TGGCGATGGTCAAGAACGA | TGATACCAAACGCGATCATCTC | 60 |
| *Osactin*  (X15865) | TGTATGCCAGTGGTCGTACCA | CCAGCAAGGTCGAGACGAA | 121 |
| *CsLOB1*  (XM_006485865) | TCCACCAACCGAACCATACA | GGCACTTGCTTCATAGACCAT | 136 |
| *CsEF1α*  (XM_006485840) | GTAACCAAGTCTGCTGCCAAG | GACCCAAACACCCAACACATT | 127 |
| *CsTFIIAγ* (KU377726) | TGAAGAGCAAGGTCTCCATTAAGG | TTCCTCACTCTTGAACAAAGCATCT | 101 |
| *CaTFIIAγ* (KU163013) | GACGATTGGGATGTGCTTGACT | ACTTGTCAAACTGAACGGGAACTT | 101 |
| *Caactin* (AY572427) | CTTCAATGTTCCGGCCATGT | TCACACCATCACCAGAGTCCAA | 101 |
| *Slactin* (U60478) | ATGGAAGCTGCAGGAATCCA | CCACCACTGAGCACAATGTTTC | 101 |

**Supplementary Table 3.** Single nucleotide polymorphisms in the Os*TFIIAγ5* coding region of the 3,024 rice varieties from the International Rice Genebank Collection Information System (IRGCIS, <http://www.oryzasnp.org/iric-portal/index.zul>).

| Varieties | IRGC^a^ ID | IRIS^b^ ID | Subpopulation | 437499^c^ | 437500^c^ | The 39^th^ amino acid |
| --- | --- | --- | --- | --- | --- | --- |
| OR 117-8 | 39680-2 | 313-11242 | ind2 | A | G | E |
| PSBRC82 |  | CX358 | ind1B | A | G | E |
| DUDHSAR | 26458-1 | 313-10980 | admix | A | G | E |
| IR62266-42-6-2 |  | CX234 | indx | A | G | E |
| BG 300 |  | CX34 | indx | A | G | E |
| JABOR SAIL | 66831-1 | 313-8410 | aus | A | G | E |
| CHUNGUR BALI | 25855-1 | 313-9422 | aus | A | G | E |
| AUS 439 | 29221-1 | 313-8641 | aus | A | G | E |
| BINNAFUL | 53479-1 | 313-11481 | aus | A | G | E |
| BONGEZA | 53483-1 | 313-11482 | aus | A | G | E |
| BOTESHSHORE | 53484-1 | 313-11483 | aus | A | G | E |
| EJALI | 53500-1 | 313-11484 | aus | A | G | E |
| SIRHANTI | 53930-1 | 313-11492 | aus | A | G | E |
| UPRH 58 | 61525-1 | 313-11618 | aus | A | G | E |
| D 204-1 | 6445-1 | 313-10534 | aus | A | G | E |
| DJ 69 | 8482-1 | 313-10592 | aus | A | G | E |
| DD 126 | 8667-1 | 313-10598 | aus | A | G | E |
| ARC 10843 | 12653-1 | 313-10673 | aus | A | G | E |
| LAL SAR | 16185-1 | 313-10735 | aus | A | G | E |
| RERM BILASH | 16273-2 | 313-10736 | aus | A | G | E |
| PANKHIRAJ | 24139-1 | 313-10930 | aus | A | G | E |
| SUNGA WALA | 26802-1 | 313-10987 | aus | A | G | E |
| CHAKILA | 27540-1 | 313-11014 | aus | A | G | E |
| HANPA | 27547-2 | 313-11015 | aus | A | G | E |
| KACHILON | 27555-1 | 313-11016 | aus | A | G | E |
| LOROI | 27567-1 | 313-11017 | aus | A | G | E |
| PORANG | 27577-1 | 313-11019 | aus | A | G | E |
| AUS 278 | 29068-1 | 313-11052 | aus | A | G | E |
| AUS 282 | 29072-1 | 313-11053 | aus | A | G | E |
| AUS 295 | 29083-1 | 313-11054 | aus | A | G | E |
| AUS 299 | 29087-1 | 313-11055 | aus | A | G | E |
| AUS 308 | 29096-1 | 313-11057 | aus | A | G | E |
| AUS 329 | 29116-1 | 313-11058 | aus | A | G | E |
| AUS 449 | 29230-1 | 313-11061 | aus | A | G | E |
| BAZAIL 975 | 32816-2 | 313-11123 | aus | A | G | E |
| NATEL BORO | 34749-1 | 313-11163 | aus | A | G | E |
| BHUT MURI | 34861-1 | 313-11166 | aus | A | G | E |
| M 142 | 35054-1 | 313-11171 | aus | A | G | E |
| SADA AUS | 35117-1 | 313-11173 | aus | A | G | E |
| KHAMA 1183 | 37685-2 | 313-11216 | aus | A | G | E |
| ARC 7425 | 42538-1 | 313-11291 | aus | A | G | E |
| KALABOKRI | 43872-1 | 313-11324 | aus | A | G | E |
| AJAYA |  | CX96 | ind1B | A | G | E |
| CX418 |  | CX418 | ind1B | A | G | E |
| CX424 |  | CX424 | ind1B | A | G | E |
| CHANDARHAT | 25845-1 | 313-9368 | aus | A | G | E |
| KALIA | 34699-1 | 313-9626 | aus | A | G | E |
| LENJA MURALI | 66815-1 | 313-9661 | aus | A | G | E |
| LALSAITA | 43915-1 | 313-8789 | aus | A | G | E |
| NOROI | 31611-1 | 313-8864 | aus | A | G | E |
| SERIBU | 71615-1 | 313-8987 | index | A | G | E |
| HATEA | 86973-1 | 313-12141 | aus | A | G | E |
| DB 3 | 8361-1 | 313-10587 | aus | A | G | E |
| DJ 29 | 8505-1 | 313-10594 | aus | A | G | E |
| DL 5 | 8593-1 | 313-10595 | aus | A | C | D |
| UCP 122 | 8794-1 | 313-10603 | aus | A | G | E |
| DV 86 | 8840-1 | 313-10605 | aus | A | G | E |
| DEVARASI | 16173-1 | 313-10734 | aus | A | G | E |
| ARC 7001 | 20436-1 | 313-10849 | aus | A | G | E |
| ARC 7336 | 20606-1 | 313-10852 | aus | A | G | E |
| INDIA DULAR | 26070-1 | 313-10969 | aus | A | G | E |
| LAKHSMI DIGHA | 26390-2 | 313-10976 | aus | A | G | E |
| BORA DIGA | 26439-1 | 313-10979 | aus | A | G | E |
| BOTESWAR 2 | 27536-1 | 313-11013 | aus | A | G | E |
| AUS 219 | 29031-1 | 313-11049 | aus | A | G | E |
| AUS 233 | 29036-1 | 313-11050 | aus | A | G | E |
| AUS 242 | 29040-1 | 313-11051 | aus | A | G | E |
| AUS 301 | 29089-1 | 313-11056 | aus | A | G | E |
| AUS 344 | 29131-1 | 313-11059 | aus | A | G | E |
| HIJOL DIGA | 31655-1 | 313-11112 | aus | A | G | E |
| KORTIK KAIKA | 31841-1 | 313-11116 | aus | A | G | E |
| BAZAIL 980 | 32817-2 | 313-11124 | admix | A | G | E |
| KALAI | 33151-2 | 313-11131 | index | A | G | E |
| MANSAT 3 | 33341-1 | 313-11134 | index | A | G | E |
| BAK TULSI | 34831-1 | 313-11164 | aus | A | G | E |
| RANI BHOG | 35109-1 | 313-11172 | aus | A | G | E |
| NARIKEL BADI | 37550-1 | 313-11213 | aus | A | G | E |
| ARC 15129 | 41938-1 | 313-11277 | aus | A | G | E |
| CX419 |  | CX419 | ind1B | A | G | E |
| CX422 |  | CX422 | ind1B | A | G | E |
| GORIA | 58736-1 | 313-11557 | aus | A | G | E |
| ARC 7255 | 12343-2 | 313-10668 | index | A | G | E |
| GHAIYA | 16286-1 | 313-10737 | aus | A | G | E |
| ARC 5756 | 20220-2 | 313-10845 | aus | A | G | E |
| ARC 11822 | 21677-1 | 313-10873 | aus | A | G | E |
| SADUMONI | 25919-1 | 313-10964 | aus | A | G | E |
| DHARIAL | 34034-1 | 313-11154 | aus | A | G | E |
| LAL TAURA | 35017-1 | 313-11170 | aus | A | G | E |
| ZARBASAIL | 37346-1 | 313-11210 | aus | A | G | E |
| AUS PADDY (RED) | 44978-1 | 313-11348 | aus | A | G | E |
| CHILE BORO | 45297-2 | 313-11353 | aus | A | G | E |
| MADHUKAR |  | CX155 | ind2 | A | G | E |
| CX425 |  | CX425 | ind1B | A | G | E |
| WIR 1391 | 51605-1 | 313-9963 | aus | A | G | E |
| UCP 41 | 8742-1 | 313-10600 | aus | A | G | E |
| SAITA | 31618-1 | 313-11111 | aus | A | G | E |
| KARIA | 6702-1 | 313-10545 | aus | A | G | E |
| DJ 47 | 8497-1 | 313-10593 | aus | A | G | E |
| KADA CHOPA | 34954-1 | 313-11168 | aus | A | G | E |
| JASURE | 43860-1 | 313-11322 | aus | A | G | E |
| DHALA AMAN 973 | 32861-1 | 313-11125 | admix | A | G | E |
| ARC 14901 | 41811-1 | 313-11274 | aus | A | G | E |
| IRBB60 |  | CX126 | ind1B | A | G | E |
| KASALATH |  | CX227 | aus | A | G | E |
| SLO 19 | 35157-1 | 313-11174 | aus | A | G | E |

^a^International Rice Genebank Collection.

^b^International Rice Information System.

^c^Physical location on rice chromosome 5.

**Supplementary Table 4.** *Xanthomonas* TALE binding elements were overlapped or closed to the TATA box of their target gene’s promoters

| Organism^a^ | TALE | Host  plant | Target  gene | Target DNA sequence  (EBE region and **TATA** box) | EBE distance  to ATG |
| --- | --- | --- | --- | --- | --- |
| *Xoo*  PXO99^A^ | pthXo1/  tal2b | rice | *Xa13/Os8N3/*  *SWEET11*^c^ | ATGCATCTCCCCCTACTGTACACCACC…(19)…C**TATAT**A | -251 ~ -227 |
| *Xoo* | pthXo4 | rice | *Xa13/Os8N3/*  *SWEET11*^d^ | CTA**TATAA**ACACTGAGCCA | -205 ~ -189 |
| *Xoo* | pthXo5 | rice | *Xa13/Os8N3/*  *SWEET11*^d^ | CTA**TATAA**ACACTGAGCCATGG | -205 ~ -186 |
| *Xoo*  PXO99^A^ | pthXo6/  tal5b | rice | *OsTFX1* ^c^ | C**TATAA**AAGGCCCTCACCAACCCATC | -136 ~ -113 |
| *Xoo*  PXO99^A^ | pthXo7/  tal1 | rice | *OsTFIIAγ1* ^c^ | A**TATAA**TCCCCAAATCCCCTCCTCC | -469 ~ -447 |
| *Xoo*  PXO99^A^ | tal9a | rice | *OsHEN1* ^c^ | G**TATTA**T…(52)…TTCCCTTCCCTAAACCCCACTTC | -206 ~ -186 |
| *Xoo*  PXO99^A^ | AvrXa27/  tal9c | rice | *Xa27* ^c^ | C**TATAA**ATAGAAGAAGAGACCCATAG | -87 ~ -70 |
| *Xoo*  PXO99^A^ | AvrXa23/  tal9b | rice | *Xa23* ^c^ | TTCCGAAACATCTTCCTCCCGCATCACTAA…(9)…C**TATAA**A | -125 ~ -97 |
| *Xoo*  PXO86 | AvrXa10 | rice | *Xa10* ^c^ | A**TATAT**ACACACGTTCACT | -111 ~ -95 |
| *Xoo*  JXO1 | pthXo2 | rice | *Xa25* ^c^ | A**TATAA**AGCACCACAACTCCCTTC | -225 ~ -204 |
| *Xoo*  PXO61 | pthXo3 | rice | *Os11N3/*  *SWEET14* ^c^ | CTA**TATAA**ACCCCCTCCAACCAGGTGCTAAGC | -261 ~ -232 |
| *Xoo*  PXO86 | AvrXa7 | rice | *Os11N3/*  *SWEET14* ^c^ | A**TATAA**ACCCCCTCCAACCAGGTGCTAAG | -259 ~ -233 |
| *Xoo*  BAI3^R^ | talC | rice | *Os11N3/*  *SWEET14* ^c^ | CATGCATGTCAGCAGCTGGTCATG…(35)…A**TATAA**A | -318 ~ -297 |
| *Xoo*  AXO1947 | talC | rice | *Os11N3/*  *SWEET14* ^c^ | CATGCATGTCAGCAGCTGGTCATG…(35)…A**TATAA**A | -318 ~ -297 |
| *Xoo*  MAI1 | tal5 | rice | *Os11N3/*  *SWEET14* ^c^ | A**TATAA**A…(17)…CTAAGCTCATCAAGCCTTCAA | -235 ~ -217 |
| *Xoo*  PXO86 | AvrXa7 | rice | *Xa7*^b c^ | CTA**TATAA**ACCCCCTCCAACCAGGTGCTA |  |
| *Xoc*  BLS256 | tal1c | rice | *OsHEN1* ^d^ | G**TATTA**T…(42)…TCCCCCTCGCTTCCCTTC | -216 ~ -201 |
| *Xoc*  BLS256 | tal4c | rice | *Os06g37080* ^d^ | C**TATAA**AACCTGGACAAGCCTCTCTC | -151 ~ -128 |
| *Xoc*  BLS256 | tal2g | rice | *Os01g52130/*  *OsSULTR3;6* ^c^ | TGGCCCGTAGCCTCTCCTT…(11)…A**TATAA**A | -427 ~ -410 |
| *Xoc*  BLS256 | tal2g | rice | *Os06g46500* ^d^ | T**TATAA**T…(103)…TGGCAAGTGACCTCAGCTC | -88 ~ -71 |
| *Xca*  5 | Hax2 | Arabidopsis | *PAP1*  */At1g56650* ^c^ | CTCACATACTCACACTCTCTATAAG…(25)…C**TATAT**ATAC | -130 ~ -107 |
| *Xcv*  71-21 | AvrBs3 | pepper | *Bs3* ^c^ | TTA**TATAA**ACCTAACCATCC | -121 ~ -103 |
| *Xcv*  85-10 | AvrBs3 | pepper | *UPA20* ^c^ | TTA**TATAA**ACCTGACCCTTT | -151 ~ -134 |
| *Xcv*  85-10 | AvrBs3 | pepper | *UPA10* ^d^ | CTA**TATAA**ACCAACCCCCATC | -140 ~ -122 |
| *Xcv*  85-10 | AvrBs3 | pepper | *UPA12* ^d^ | TA**TATAA**GTAAAACTCTCCCTCTC | -152 ~ -134 |
| *Xcv*  85-10 | AvrBs3 | pepper | *UPA19* ^d^ | T**TATAT**ACACCCCTCCTCCCA | -135 ~ -117 |
| *Xcv*  85-10 | AvrBs3 | pepper | *UPA23* ^d^ | CTTA**TATAA**AACTTCCCTCTCA | -132 ~ -113 |
| *Xg*  XV444 | AvrHah1 | pepper | *Bs3* ^c^ | TA**TATAA**ACCTAACCATC | -120 ~ -104 |
| *Xcv* | AvrBs3Δrep16 | pepper | *Bs3-E* ^c^ | TTA**TATAA**ACCTCTCTA | -133 ~ -120 |
| *Xcv* | AvrBs3Δrep109 | pepper | *Bs3* ^c^ | TTA**TATAA**ACCTAACCAT | -121 ~ -105 |
| *Xcv*  82-8 | AvrBs4 | pepper | *Bs4C** ^c^ | A**TATAA**AAAATAGTCCTCTCA | -181 ~ -163 |
| *Xcc*  306 | pthA4 | citrus | *CsLOB1* ^c^ | A**TATAA**ACCCCTTTTGCCTTA | -117 ~ -99 |
| *Xcc*  A^w^ | pthAw | citrus | *CsLOB1* ^c^ | A**TATAA**ACCCCTTTTGCCTTA | -117 ~ -99 |
| *Xcc*  A* | pthA* | citrus | *CsLOB1* ^c^ | A**TATAA**ACCCCTTTTGCCTTA | -117 ~ -99 |
| *Xcc*  049 | TalC | citrus | *CsLOB* ^c^ | A**TATAA**ACCCCTTTTGCCTTA | -114 ~ -99 |
| *Xcc*  3213 | pthA | citrus | *CsLOB* ^c^ | A**TATAA**ACCCCTTTTGCCTTA | -117 ~ -99 |
| *Xcc*  306 | pthA4 | citrus | *CsSWEET1* ^c^ | A**TATAA**ACCGCTTTTGCTTCT | -149 ~ -131 |
| *Xcc*  A^w^ | pthAw | citrus | *CsSWEET1* ^c^ | A**TATAA**ACCGCTTTTGCTTCT | -149 ~ -131 |
| *Xcc* | pthA* | citrus | *CsSWEET1* ^c^ | A**TATAA**ACCGCTTTTGCTTCT | -149 ~ -131 |
| *Xcc*  A^w^ | pthAw | citrus | *CsSWEET1* ^c^ | TTATTTTACTGCGTTGCAGTT…(18)…A**TATAA**A | -188 ~ -170 |
| *Xfa*  B | pthB | citrus | *CsLOB1* ^c^ | TTCTCTA**TATAA**ACCCCTTTT | -123 ~ -105 |
| *Xfa*  C | pthC | citrus | *CsLOB1* ^c^ | TTCTCTA**TATAA**ACCCCTTTT | -123 ~ -105 |
| *Xam*  *668* | TAL20 | cassava | *MeSWEET10a* ^c^ | CTA**TATAA**ACGCTTCTCGCCCATCC | -110 ~ -91 |
| *Xg*  *153*  *Xg*  *153* | AvrHah1  AvrHah1 | tomato  tomato | *bHLH3* ^c^  *bHLH6* ^c^ | TATAGA**TATAA**GCTACCAGC  TATACAGGATATCCCTTTCATCATTA**TATAA**A | -277 ~ -260  -167 ~ -150 |

^a^*Xoo*, *Xanthomonas oryzae* pv. *oryzae*; *Xoc*, *Xanthomonas oryzae* pv. *oryzicola*; *Xca*, *Xanthomonas campestris* pv.*armoraciae*; *Xcv*, *Xanthomonas campestris* pv. *vesicatoria*; *Xg*, *Xanthomonas gardneri*; *Xcc*, *Xanthomonas citri* ssp. *citri*; *Xfa*, *Xanthomonas fuscans* ssp. *aurantifolii*; *Xam*, *Xanthomonas axonopodis* pv. *manihotis*;

^b^gene not been cloned

^c^Identified target gene

^d^Predicted target gene
