# Supplementary material for: A Conserved Basal Transcription Factor Is Required for the Function of Diverse TAL Effectors in Multiple Plant Hosts
Source: Front Plant Sci. 2017 Nov 7;8:1919. doi: 10.3389/fpls.2017.01919 (PMC5681966; doi:10.3389/fpls.2017.01919)
Supplement: Supplementary file 2 [file Image_1.PDF]

```
TALE             1       10        20        30        40        50        60        70        80        90       100       110       120       130 134
                 ---------+---------+---------+---------+---------+---------+---------+---------+---------+---------+---------+---------+---------+----
PthXo7/Tal1      SIVAQLSRPDPALAALTNDHLVALACLGGRPALDAVKKGLPHAPELIRRINRRIPERTSHRVPDLAHVVRVLGFFQSHSHPAQAFDDAMTQFEMSRHGLVQLFRRVGVTEFEARYGTLPPASQRWDRILQASGM   Xoo strain (PXO99)

TFBTal9b/TFB11   ..........................................................................................Q..L..................................   Xoc strain (RH3)

TFBi/PthA1       ............................................A..K.T............A.H.Q.........C...............G......L..........L...S...................   Xcc strain (X02-007)
TFBii            ............................................A..K.T.......A....A.H.Q.........C...............G......L..........L...S...................
TFBiii           ............................................A..K.T............A.H.Q......L..C...............G......L..........L...S...................

TFBc             .................................................V....G.......A.Y.......E.........H...E...........................G..........H........   Xcv strain (23-1)
TFBd             .................................................V....G.......A.Y.Q.....E.........H...E.........N..L............T.G..........H........
TFBa             .............T...................................V....G.......A.Y.Q.....E.........H...E...........................C..................
TFBb             .................................................V....G.......A.Y.Q.....E...S..Q..................................SG..........H........
TFBe             .................................................V....G.......A.Y.Q.....E......Q.....................P............S..................
```

FIGURE S1 | Amino acid sequence alignment of the TFB motifs of TALEs from *Xanthomonas oryzae* pv. *oryzae* (*Xoo*), *Xanthomonas oryzae* pv. *oryzicola* (*Xoc*), *Xanthomonas citri* pv. *citri* (*Xcc*), and *Xanthomonas campestris* pv. *vesicatoria* (*Xcv*).

A

```
Hongkong kumquat  ATGGCGACGTTTGAGCTGTATCGCAGGTCGACGATTGGGATGTGCTTAACCGAGACTTTA
Grapefruit        ATGGCGACGTTTGAGCTGTATCGCAGGTCGACGATTGGGATGTGCTTAACCGAGACTTTA
Sweet orange      ATGGCGACGTTTGAGCTGTATCGCAGGTCGACGATTGGGATGTGCTTAACCGAGACTTTA
Trifoliate orange ATGGCGACGTTTGAGCTGTATCGCAGGTCGACGATTGGGATGTGCTTAACCGAGACTTTA
                  ************************************************************

Hongkong kumquat  GACGAGATGGTCCAAAACGGTACGCTTACTCCCGAGCTCGCTATCCAGGTTCTCGTCCAG
Grapefruit        GACGAGATGGTCCAAAACGGTACGCTTACTCCCGAGCTAGCTATCCAGGTTCTCGTCCAG
Sweet orange      GACGAGATGGTCCAAAACGGTACGCTTACTCCCGAGCTAGCTATCCAGGTTCTCGTCCAG
Trifoliate orange GACGAGATGGTCCAAAACGGTACGCTTACTCCCGAGCTAGCTATCCAGGTTCTCGTCCAG
                  ************************************** *********************

Hongkong kumquat  TTCGATAAGTCTATGACAGAAGCACTAGAAACTCAGGTGAAGAGCAAGGTCTCCATTAAG
Grapefruit        TTCGATAAGTCTATGACAGAAGCACTAGAAACTCAGGTGAAGAGCAAGGTCTCCATTAAG
Sweet orange      TTCGATAAGTCTATGACAGAAGCACTAGAAACTCAGGTGAAGAGCAAGGTCTCCATTAAG
Trifoliate orange TTCGATAAGTCTATGACAGAAGCACTAGAAACTCAGGTGAAGAGCAAGGTCTCCATTAAG
                  ************************************************************

Hongkong kumquat  GGGCATCTGCACACCTACAGGTTCTGTGACAATGTGTGGACATTCATCTTGCAAGATGCT
Grapefruit        GGGCATCTGCACACCTACAGGTTCTGTGACAATGTTTGGACATTCATCTTGCAAGATGCT
Sweet orange      GGGCATCTGCACACCTACAGGTTCTGTGACAATGTTTGGACATTCATCTTGCAAGATGCT
Trifoliate orange GGGCATCTGCACACCTACAGGTTCTGTGACAATGTTTGGACATTCATCTTGCAAGATGCT
                  ********************************** *************************

Hongkong kumquat  TTGTTCAAGAGTGAGGAATTACAAGAGACCGTCGGTAGGGTGAAAATAGTGGCATGTGAC
Grapefruit        TTGTTCAAGAGTGAGGAATTACAAGAGACCGTTGGTAGGGTGAAAATAGTGGCATGTGAC
Sweet orange      TTGTTCAAGAGTGAGGAATTACAAGAGACCGTTGGTAGGGTGAAAATAGTGGCATGTGAC
Trifoliate orange TTGTTCAAGAGTGAGGAATTACAAGAGACCGTTGGTAGGGTGAAAATAGTGGCATGTGAC
                  ******************************* ****************************

Hongkong kumquat  TCAAAGCTGCTATCACAATAA
Grapefruit        TCAAAGCTGCTATCACAATAA
Sweet orange      TCAAAGCTGCTATCACAATAA
Trifoliate orange TCAAAGCTGCTATCACAATAA
                  *********************
```

B

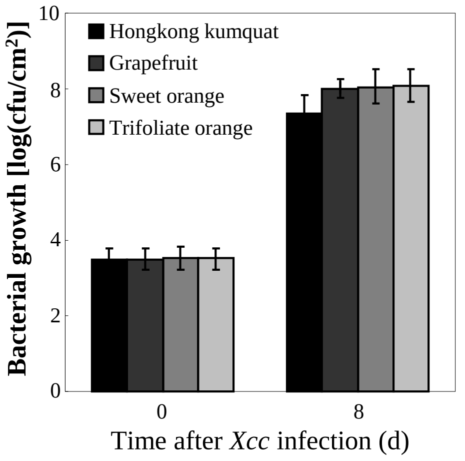

FIGURE S2 | Sequence alignment of Cs*TFIIAγ* gene coding regions and response to *Xcc* of different *Citrus* species. **(A)** Cs*TFIIAγ* genes from different *Citrus* species encode identical protein, although they have nucleotide polymorphisms. **(B)** Different *Citrus* species showed a similar level of susceptibility to *Xcc* strain X02-007.

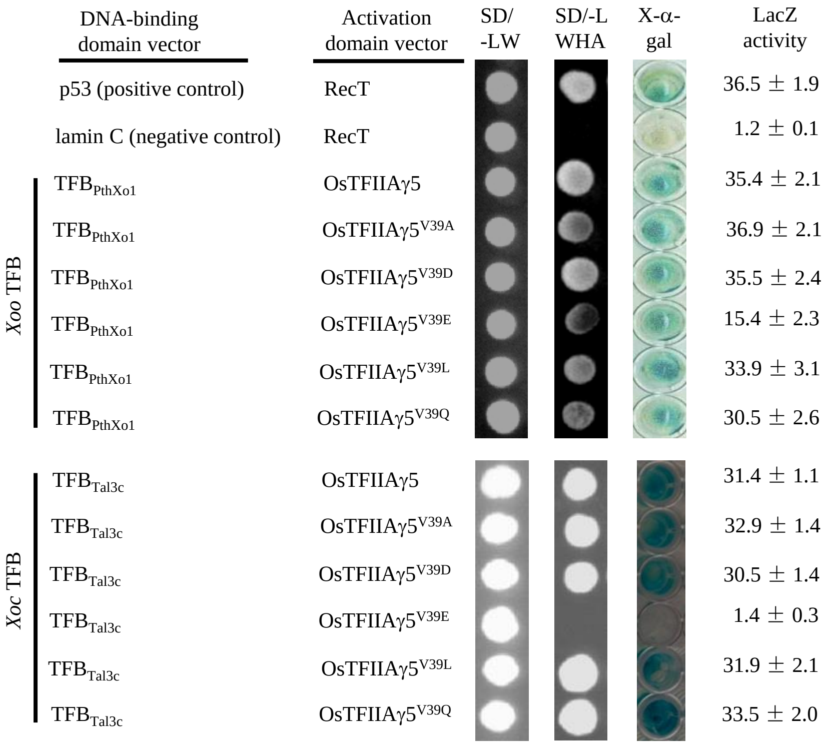

FIGURE S3 | Interactions between the TFB motif of *Xoo* TALE PthXo1 or *Xoc* TALE Tal3c and mutated OsTFIIAγ5s as determined by yeast two-hybrid. The interactions were assessed by growth of yeast cells on synthetic defined premixes (SD) medium lacking (-) leucine (L), tryptophan (W), histidine (H), and adenine (A). The interaction with different strength based on the analysis of LacZ activity.

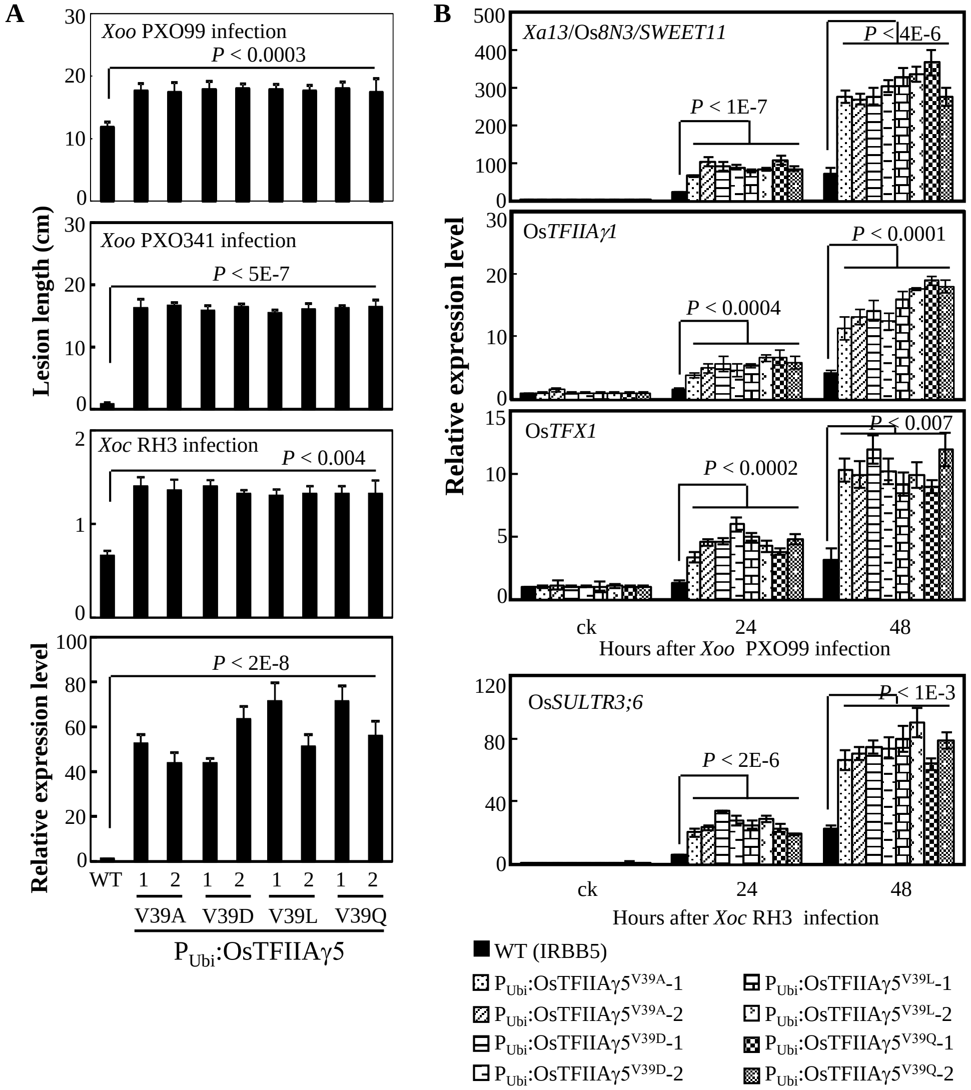

FIGURE S4 | Effects of overexpressing Os*TFIIAγ5* mutants in rice responses to *Xoo* and *Xoc*. **(A)** Transgenic rice plants carrying Os*TFIIAγ5* mutant were susceptible to *Xoo* stains PXO99 and PXO341 and *Xoc* strain RH3 compared to wild type (WT), which carries the mutated Os*TFIIA*γ5^{V39E}. T1 Plants were inoculated with *Xoo* at the booting stage and *Xoc* at the tillering stage. Data represent mean (total 20 to 25 leaves from four plants) ± standard deviation. **(B)** Expression of disease susceptibility genes *Xa13/Os8N3/SWEET11*, Os*TFIIAγ1* and Os*TFX1* in rice after infection of PXO99 and *OsSULTR3;6* after infection of RH3. Bar represents mean (three replicates) ± standard deviation. The corresponding *P* values were determined using Student's *t*-test (two tailed) comparing data from the WT and transgenic plants.

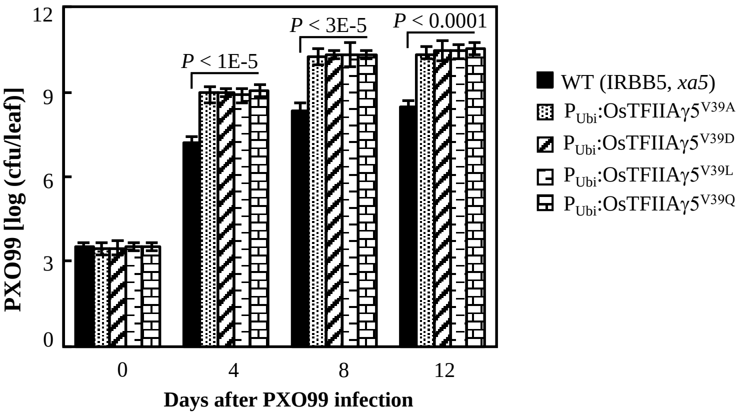

FIGURE S5 | Growth of the *Xoo* strain PXO99 in the leaves of transgenic plants (T1 generation) overexpressing Os*TFIIAγ5* mutants. cfu, colony-forming unit. Bar represents mean (three replicates) ± standard deviation. The corresponding *P* values were determined using Student's *t*-test (two tailed) comparing data from the WT and transgenic plants.

	OsTFIIAγ5	OsTFIIAγ5^{V39E}	OsTFIIAγ1	CsTFIIAγ	SlTFIIAγ	CaTFIIAγ
OsTFIIAγ5	100/100	99/99	94/88	93/89	91/86	91/86
OsTFIIAγ5^{V39E}		100/100	93/87	92/88	90/85	90/85
OsTFIIAγ1			100/100	93/83	93/86	93/86
CsTFIIAγ				100/100	95/92	95/92
SlTFIIAγ					100/100	100/100
CaTFIIAγ						100/100

FIGURE S6 | Pairwise comparison of amino acid similarity/identity (%) among rice, citrus, pepper and tomato TFIIAγs. Rice OsTFIIAγ5 (accession number in protein database or GenBank of National Center for Biotechnology Information: AAO33769), OsTFIIAγ5^{V39E} (AAV53715), and OsTFIIAγ1 (NP_001045526); citrus CsTFIIAγ (XP_006433782); tomato SlTFIIAγ (XP_004252721); pepper CaTFIIAγ (KU163013).

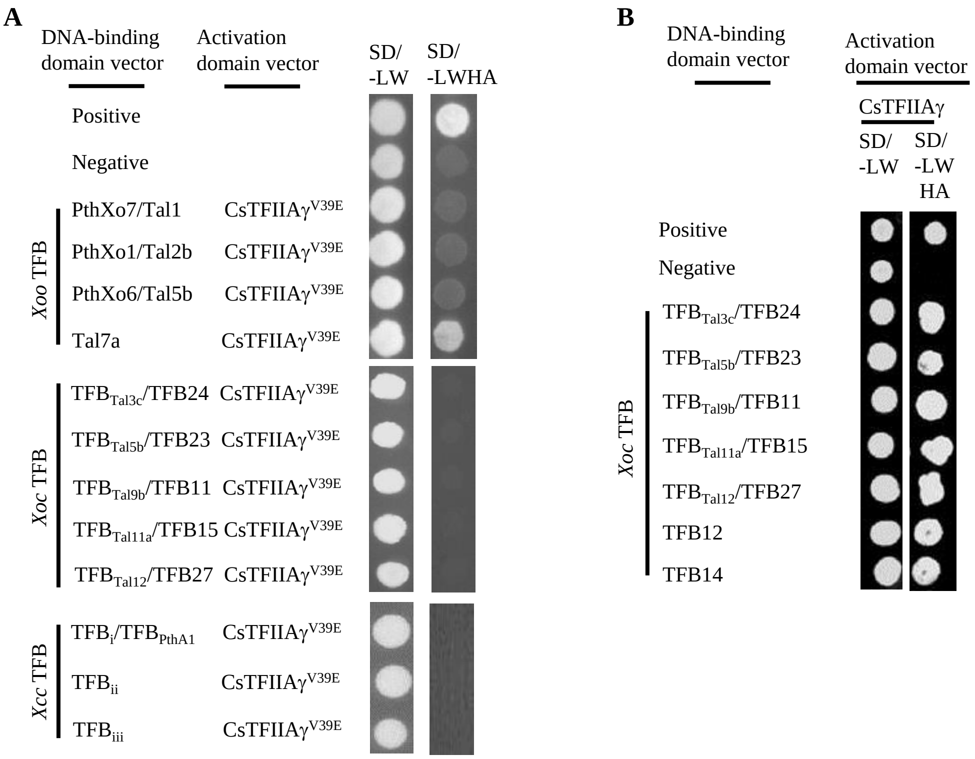

FIGURE S7 │Analysis of the interaction between CsTFIIAγ^{V39E} and TFB motifs of different TALEs from *Xoo*, *Xoc* and *Xcc* by yeast two-hybrid assays. The interactions were assessed by growth of yeast cells on synthetic defined premixes (SD) medium lacking (-) leucine (L), tryptophan (W), histidine (H), and adenine (A). **(A)** The TFB motifs of *Xoo*, *Xoc* and *Xcc* TALEs could not interact with CsTFIIAγ^{V39E}. **(B)** Interactions between the TFB motifs of TALEs from *Xoc* and CsTFIIAγ.

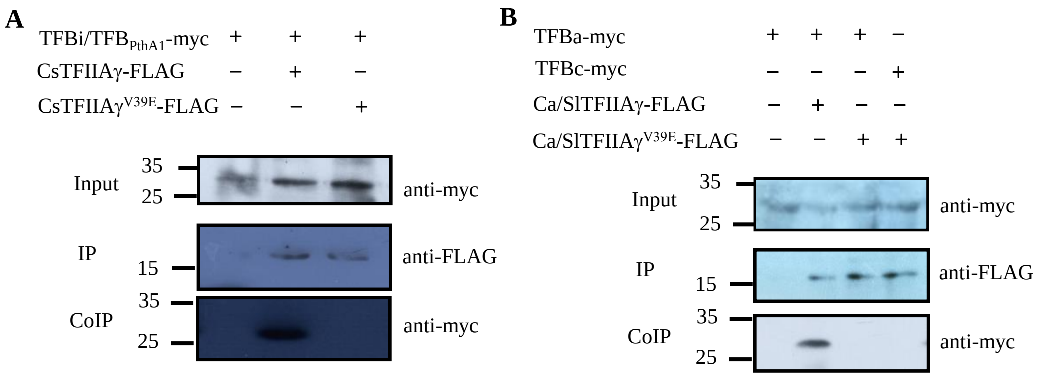

FIGURE S8 │The interactions between mutated TFIIAγ and TFB motifs of TALEs analyzed in *Nicotiana benthamiana* leaf cells analyzed by coimmunoprecipitation (CoIP) assay. Proteins before (input) and after immunoprecipitation (IP) were detected with anti-myc and anti-FLAG antibodies. **(A)** The myc-labelled TFB motifs of TALEs from *Xcc* not interacted with FLAG-labelled CsTFIIAγ^{V39E}. **(B)** The myc-labelled TFB motifs of TALEs from *Xcv* not interacted with FLAG-labelled CaTFIIAγ^{V39E} or SlTFIIAγ^{V39E}.

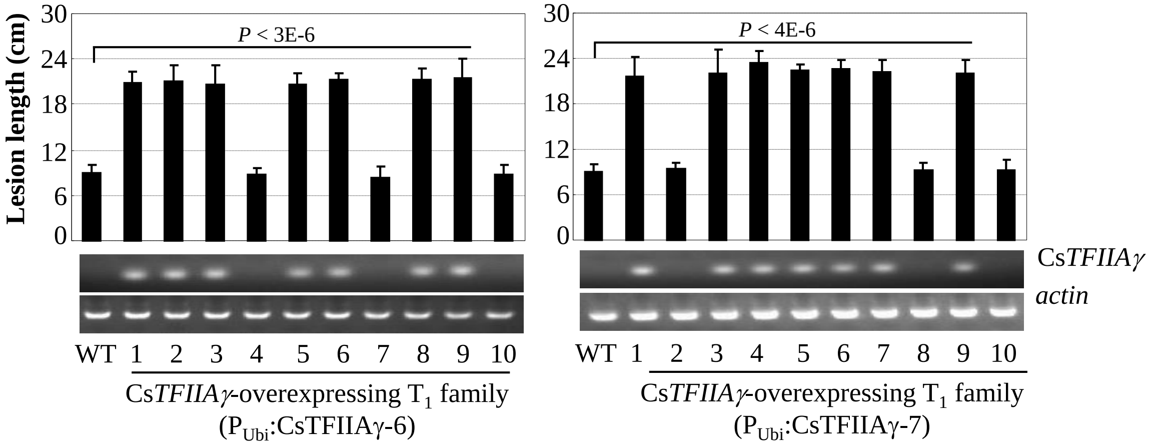

FIGURE S9 | The enhanced susceptibility of *CsTFIIAγ* overexpressing (P_{Ubi}:CsTFIIAγ) plants to *Xoo* strain PXO99 was associated with ectopic transcription of Cs*TFIIAγ*. Each bar represents mean (5 to 10 leaves from one plant) ± standard deviation. The corresponding *P* values were determined using Student's *t*-test (two tailed) comparing data from the WT and transgenic plants.

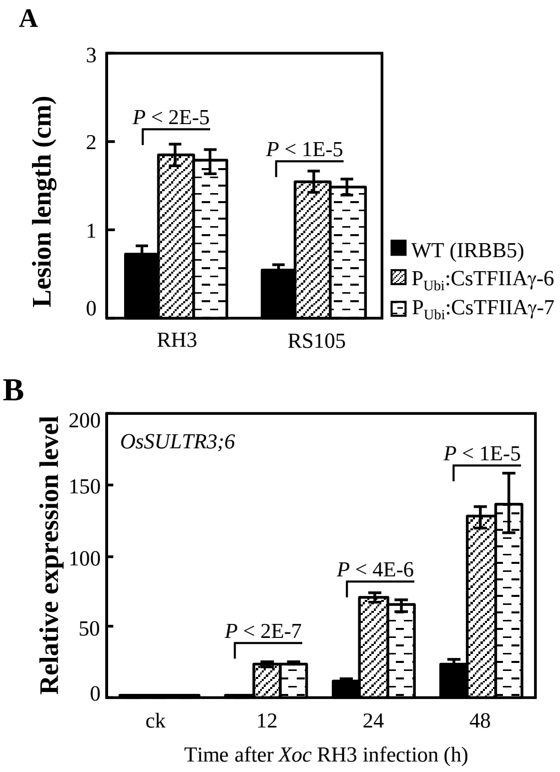

FIGURE S10 | Effect of overexpressing *CsTFIIAγ* (P_{Ubi}:CsTFIIAγ) in rice response to *Xoc*. **(A)** transgenic rice plants were susceptible to *Xoc* stains RH3 and RS105 compared to WT. Plants were inoculated with *Xoc* at the tillering stage. Data represent mean (5 to 10 replicates from one plant) ± standard deviation. **(B)** Expression of disease susceptibility gene Os*SULTR3;6* in rice after infection of *Xoc* strain RH3. Bar represents mean (three replicates) ± standard deviation. The corresponding *P* values were determined using Student's *t*-test (two tailed) comparing data from the WT and transgenic plants.

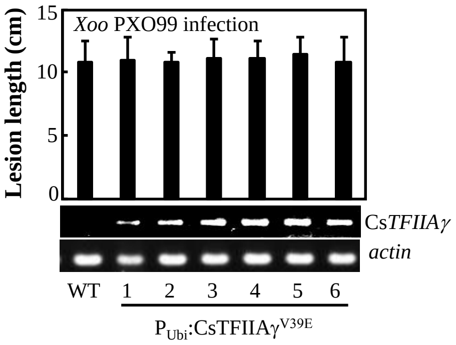

FIGURE S11 | Overexpressing of Cs*TFIIAγ*^{V39E} in rice resulted no changes to *Xoo* compared with wild-type (WT) IRBB5 carrying Os*TFIIA*γ5^{V39E}. T0 Plants were inoculated with *Xoo* PXO99 at the booting stage. Data represent mean (five to eight leaves from each plant) ± standard deviation.

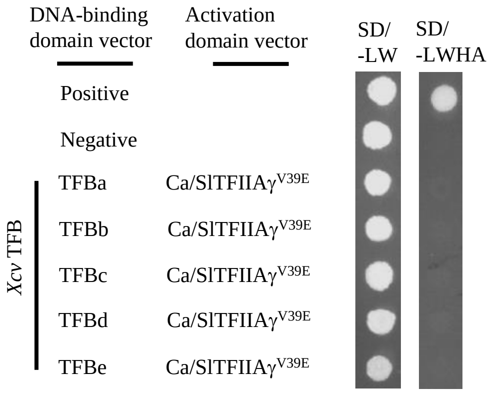

FIGURE S12 | The TFB motifs of all the five TALEs from *Xcv* strain 23-1 loss the ability of interaction with CaTFIIAγ^{V39E} and SlTFIIAγ ^{V39E} analyzed using yeast two-hybrid assay. The interactions were assessed by growth of yeast cells on synthetic defined premixes (SD) medium lacking leucine (L), tryptophan (W), histidine (H), and adenine (A).

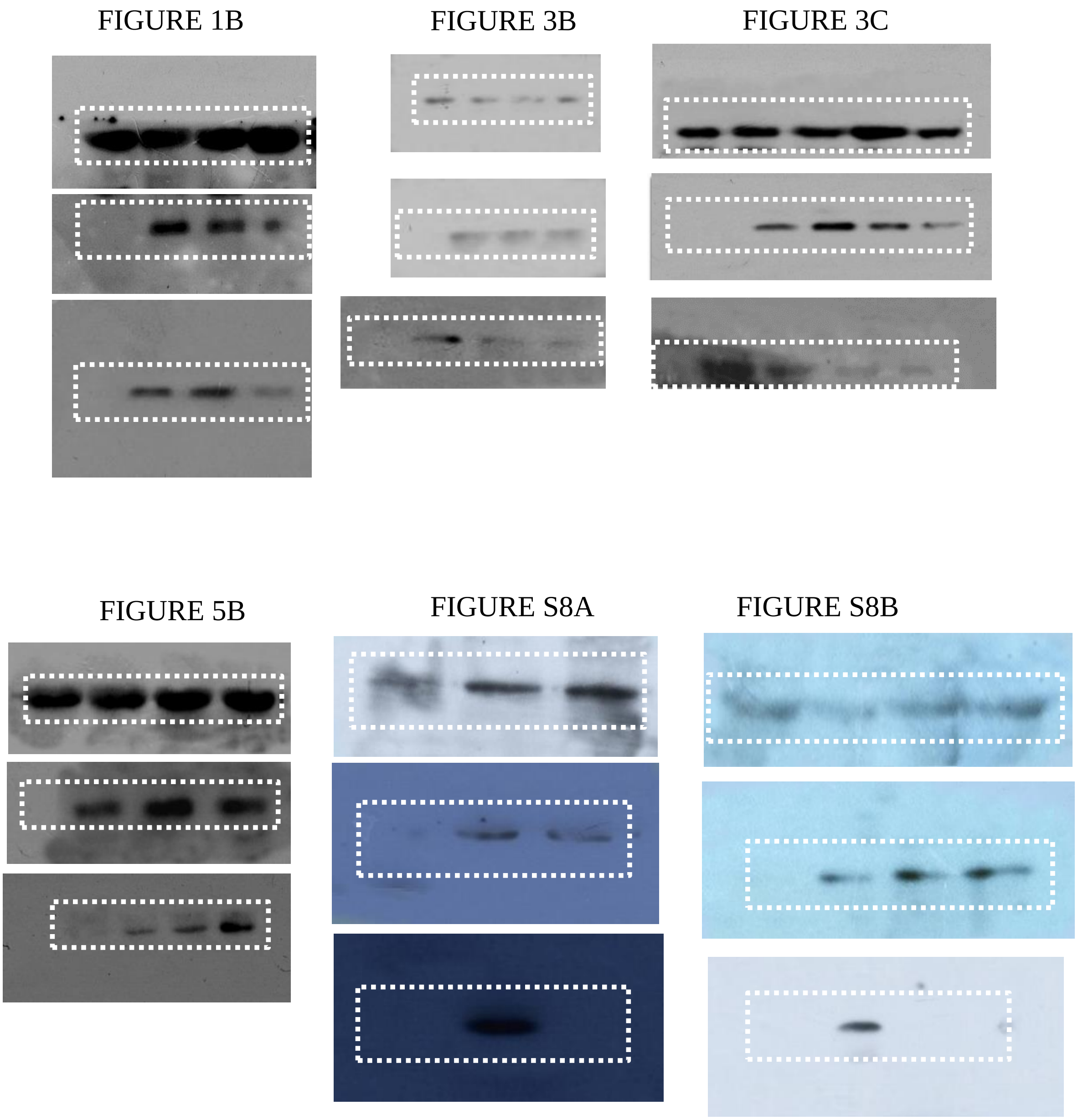

FIGURE S13 | Uncropped gel images of immunoblots.
